# The crotonylated and succinylated proteins of jujube involved in phytoplasma-stress responses

**DOI:** 10.1186/s12915-024-01917-x

**Published:** 2024-05-15

**Authors:** Liman Zhang, Huibin Wang, Chaoling Xue, Yin Liu, Yao Zhang, Zhiguo Liu, Xiangrui Meng, Mengjun Liu, Jin Zhao

**Affiliations:** 1 College of Life Science, Hebei Agricultural University, Baoding, China; 2 Hebei Key Laboratory of Plant Physiology and Molecular Pathology, Hebei Agricultural University, Baoding, China; 3 Research Center of Chinese Jujube, Hebei Agricultural University, Baoding, China

**Keywords:** Crotonylome, Enzyme activity, Inhibitor NAM, Jujube phloem, Phytoplasma-stress, Site-directed mutagenesis, Succinylome, ZjPHGPX2

## Abstract

**Background:**

Protein posttranslational modifications (PTMs) are fast and early responses to environmental changes, including pathogen infection. Jujube witches’ broom (JWB) is a phytoplasma disease causing great economic loss in jujube production. After phytoplasma infection, the transcriptional, translational, and metabolic levels in jujube were activated, enabling it to survive during phytoplasma invasion. However, no study has yet reported on PTMs in jujube. Lysine crotonylation (Kcr) and lysine succinylation (Ksu) have been popular studies in recent years and their function in plant phytoplasma-stress responses remains unclear.

**Results:**

Here, 1656 crotonylated and 282 succinylated jujube proteins were first identified under phytoplasma-stress, of which 198 were simultaneously crotonylated and succinylated. Comparative analysis revealed that 656 proteins, 137 crotonylated and 43 succinylated proteins in jujube were regulated by phytoplasma infection, suggesting that Kcr was more universal than Ksu. Kcr differentially expressed proteins (DEPs) were related to ribosomes, photosynthetic and carbon metabolism, while Ksu DEPs were mainly involved in carbon metabolism, the TCA cycle and secondary metabolite biosynthesis. The crosstalk network among proteome, crotonylome and succinylome showed that DEPs related to ribosomal, peroxidases and glutathione redox were enriched. Among them, ZjPOD51 and ZjPHGPX2 significantly increased at the protein and Kcr level under phytoplasma-stress. Notably, 7 Kcr sites were identified in ZjPHGPX2, a unique antioxidant enzyme. After inhibitor nicotinamide (NAM) treatment, GPX enzyme activity in jujube seedlings was reduced. Further, site-directed mutagenesis of key Kcr modification sites K130 and/or K135 in ZjPHGPX2 significantly reduced its activity.

**Conclusions:**

This study firstly provided large-scale datasets of Kcr and Ksu in phytoplasma-infected jujube and revealed that Kcr modification in ZjPHGPX2 positively regulates its activity.

**Supplementary Information:**

The online version contains supplementary material available at 10.1186/s12915-024-01917-x.

## Background

Protein posttranslational modifications (PTMs) are dynamic and reversible chemical modifications to proteins that affect their stability, cell signal transduction, gene expression, and enzymatic activity [1], especially fine tuning, plant responses to pathogen infection [2, 3]. Facilitated by the use of highly specific antibodies and high-resolution MS, an increasing number of novel lysine PTMs have been identified, including acetylation (Kac), crotonylation (Kcr), succinylation (Ksu) and 2-hydroxyisobutyrylation (Khib) [4–7]. The level of Khib modification increased with the infection time of *F. graminearum* in maize, and the proteins with higher Khib modification level were enriched not only in ribosome, TCA cycle but also in peroxisome, phenylpropanoid biosynthesis, jasmonic acid synthesis, the secondary metabolic processes closely related to plant disease resistance [8]. The acetylation level of *Paulownia tomentosa* changed significantly in response to phytoplasma infection [9]. Fungal pathogens promoted the susceptibility in maize through altering protein acetylation [10]. Therefore, these investigations showed that PTMs are widely involved in the regulation of interactions between plants and pathogens.

Kcr is a lysine acyl modification, the distribution and potential functions of Kcr in plant cells have also been the subject of increasing research, such as tobacco [11], rice [12], papaya [13], peanuts [14]. These researches revealed Kcr as involved in carbon fixation, amino acid biosynthesis and ribosomes, glycolysis and signal transduction. In addition, Kcr participates in abiotic and biotic stress response. In low-temperature stress, Kcr played important roles in regulating glutathione peroxidase (GPX) activity of chrysanthemum [15, 16]. Meantime, Kcr also plays important roles in plant-pathogen interactions. In *Botrytis cinerea*, 26 crotonylated proteins were identified to participate in its pathogenicity process, including signal transduction, redox homeostasis, secretion of virulence factors and plant cell wall degradation [17]. Moreover, crotonylation occurred in a variety of oxidoreductases in *B. cinerea*, and knocking out these enzymes with mutants resulted in growth retardation and impaired virulence [18].

Ksu is another conserved modification, which has been systematically identified in rice [19], wheat [20], tea [21] and pecan [22]. In pecan, Ksu proteins were mainly involved in glucose metabolism and plant-pathogen interactions. In addition, more than 40 well-reported pathogenicity-related proteins, such as MPG1 and SSB1 were identified as succinylated proteins, indicating a potential role of succinylation in the pathogenicity of *P. oryzae* [23]*.* Significantly, Ksu proteins identified in *Paulownia tomentosa* under phytoplasma-stress were found to mainly participate in peptide metabolism processes, protein folding, structural constituents of ribosomes and ATP binding [9]. Thus, research on Ksu may provide some new clues about regulatory mechanism under phytoplasma-stress.

Pathogen attack can increase ROS presence in plant, which leads to oxidative damage of proteins, nucleic acids, lipids [24, 25]. The antioxidant defense system including GPX and peroxidase (POD) is then triggered to protect the plant against ROS [26]. Phospholipids rich in unsaturated fatty acids cause a decrease in membrane fluidity, increase membrane leakage, and damage membrane proteins, thereby deactivating receptors, enzymes, and ion channels [27]. The primary cellular enzymatic defense system against damage with lipid hydroperoxide was the glutathione redox cycle with GPX. Phospholipid hydroperoxide glutathione peroxidase (PHGPX, belonging to GPX family) was a unique antioxidant enzyme, which can markedly reduce lipid hydroperoxide generated in the biomembrane [28]. Moreover, a conservative domain KWNF (S/T) KFL was the difference between PHGPX and other family members [29]. Stable expression of *LePHGPX* in tobacco afforded protection against the necrotrophic fungus *Botrytis cinerea* [30]. Besides, the increase of *TcPHGPX* expression in susceptible variety may be also related to protection against pathogens [31].

Phytoplasmas constitute a class of cell wall-free prokaryotic organisms that inhabit plant phloem tissue [32]. To date, more than 1000 plant species worldwide are known to be infected by phytoplasmas [33]. Jujube witches’ broom (JWB) is a phytoplasma disease that causes large economic losses and destructive death in the jujube industry. Phytoplasmas modulate plant morphogenesis by secreting effector proteins. Currently, several phytoplasma effectors and their homologs were demonstrated in transgenic plants. SAP11 could bind and destabilize *Arabidopsis* CINCINNATA (CIN)-related TEOSINTE BRANCHED1, CYCLOIDEA, PROLIFERATING CELL FACTORS 1 and 2 (TCP) transcription factors, thereby reducing jasmonic acid synthesis and promoting cell proliferation, leading to leaf crinkling and stem proliferation phenotypes [34]. The effector SAP54 degraded MADS-box transcription factors in transgenic *Arabidopsis* lines, producing leaf-like flowers [35]. TENGU was another effector from onion yellows phytoplasma. *Arabidopsis thaliana* lines stably expressing TENGU displayed witches’ broom, dwarfism, and flower sterility [36, 37]. In jujube, JWB phytoplasma effector ‘Zaofeng6’ induced shoot proliferation by decreasing the expression of *ZjTCP7* [38]. And, phytoplasma effectors ‘SJP1’ and ‘SJP2’ caused lateral bud outgrowth by repressing the *ZjBRC1*-controlled auxin efflux channel [39]. Moreover, the growth and development of jujube were affected at the transcriptional, translational, and metabolic levels [40–44]. However, no study has yet reported on PTMs in jujube. Therefore, this study provides a large-scale dataset of lysine crotonylation and succinylation and selects key proteins to identify key crotonylation modification sites, providing some new clues for elucidating the molecular mechanism in jujube-phytoplasma interaction.

## Results

### Identification of total crotonylated and succinylated proteins in jujube

**Fig. 1:**
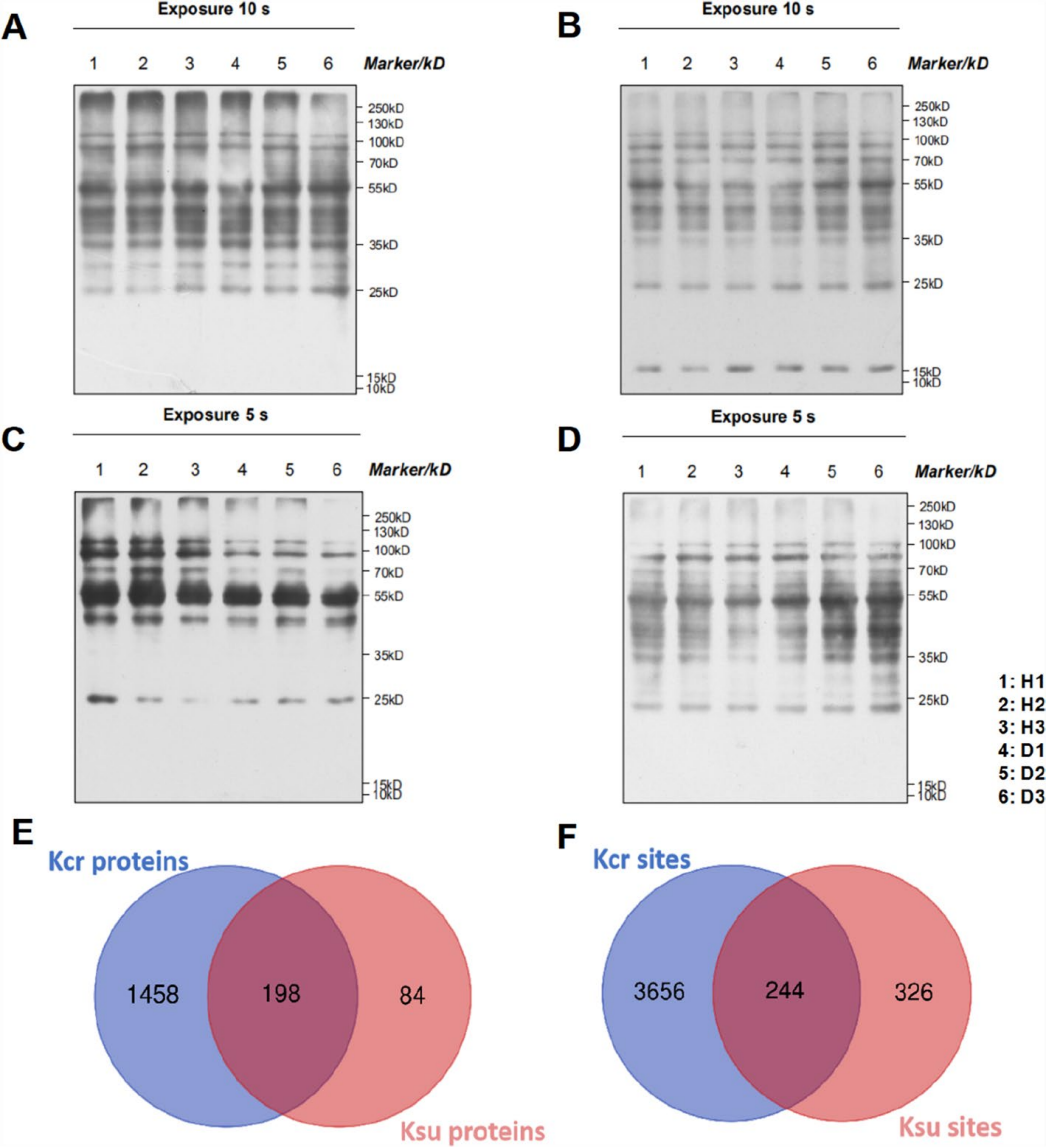
Profile of identified crotonylated and succinylated proteins and sites in jujube under phytoplasma stress. **A** WB with pan anti-2-hydroxyisobutyryllysine antibody. H1, H2 and H3 were three healthy samples; D1, D2 and D3 were three diseased samples. **B** WB with pan anti-acetyllysine antibody. **C** WB with pan anti-succinyllysine antibody. **D** WB with pan anti-crotonyllysine antibody. **E** The statistical analysis of the overlap between the Kcr and Ksu proteins. **F** The statistic of the overlap between the Kcr and Ksu sites

To explore the distribution characteristics of lysine modifications in jujube phloem proteome under phytoplasma stress, the four modified pan-antibodies were used to detect modification signals from the healthy and diseased phloem by western blotting (WB) (Fig. 1, Additional file 1: Fig. S1, Additional file 2: Fig. S2). Among them, Kcr and Ksu modifications have the most significant differences between materials, indicating that Kcr and Ksu were widespread in jujube proteins after phytoplasma infection (Fig. 1C, D). In this study, 3900 crotonylated sites in 1656 proteins and 570 succinylated sites in 282 proteins were identified and 244 sites of 198 proteins were regulated simultaneously by these two kinds of modifications (Fig. 1E, F). The mass errors and lengths of all identified peptides were examined, their mass errors were found as ≤ 5 ppm (Additional file 3: Fig. S3A, D), and the lengths of the identified peptides varied from 7 to 19 amino acid residues (Additional file 3: Fig. S3B, E), which were consistent with the properties of trypsin peptides. In addition, the lower relative standard deviation (RSD) values among the repeated samples indicated that the data had excellent repeatability (Additional file 3: Fig. S3C, F).

**Fig. 2:**
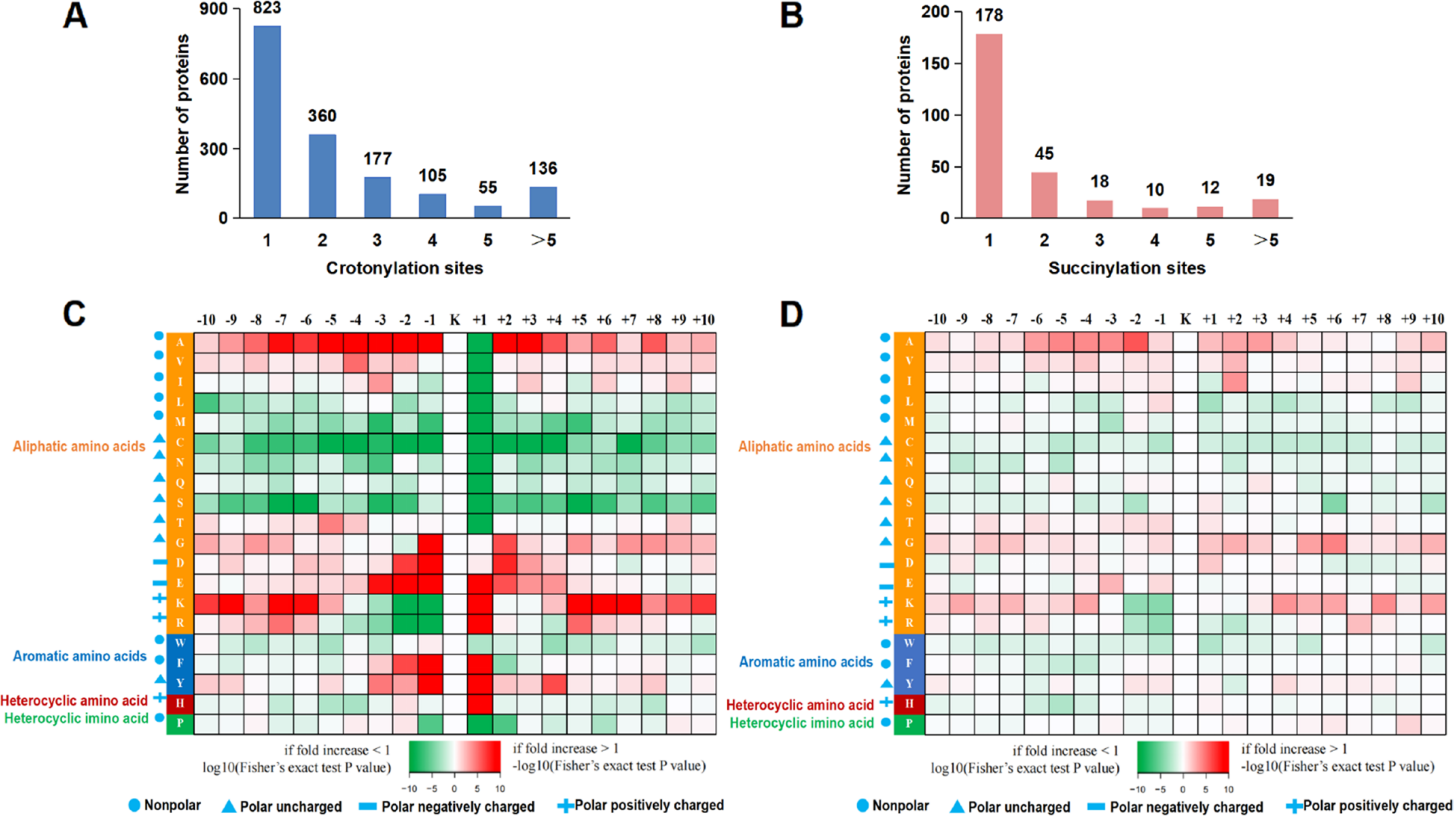
Distribution and amino acid compositions of the Kcr and Ksu sites. **A** Distribution of Kcr peptides in one protein. **B** Distribution of Ksu peptides in one protein. **C** The intensity map of crotonylation motif shows the relative abundance of ± 10 amino acids flanking the crotonylated lysine site. The colors in the intensity map mean the log10 (the frequencies within crotonyl-21-mers/the frequencies within non-crotonyl-21-mers) (red, enrichment; green, depletion); **D** The intensity map of succinylation motif shows the relative abundance of ± 10 amino acids flanking the succinylated lysine site. The colors in the intensity map mean the log10 (the frequencies within succinyl-21-mers/the frequencies within non-succinyl-21-mers) (red, enrichment; green, depletion)

A distribution analysis of the Kcr sites showed that 49.7% of the lys-crotonylated proteins had only one crotonylation site, 21.7% had two sites, 10.7% had three sites, and the rest had four sites or more than four sites; the average crotonylation degree was 2.35 (Fig. 2A). Proteins with multiple modification sites were involved in redox homeostasis, protein processing, endocytosis, carbon metabolism and phloem development. ZjSEOB2 (protein SIEVE ELEMENT OCCLUSION B-like) had 18 crotonylation sites, which were involved in phloem development and assimilate transport (Additional file 4: Table. S1). The Kcr level of ZjSEOB2 decreased significantly in diseased jujube, indicating that ZjSEOB2 may play crucial roles in the jujube-phytoplasma interaction.

For the succinylation events, 570 Ksu sites of 282 proteins were identified with an average succinylation degree of 2.02. A total of 178 proteins had one Ksu site, and 19 proteins had more than five Ksu sites (Fig. 2B). Proteins with multiple modification sites were involved in oxidative phosphorylation, carbon metabolism and TCA cycle. Some Ksu proteins were involved in glycolysis/gluconeogenesis, pyruvate metabolism and amino acid synthesis and degradation. In addition, heat shock 70 kDa protein (HSP70) had 6 Ksu sites, which were related to the RNA degradation process (Additional file 5: Table. S2).

These results provide a comprehensive overview of the crotonylation and succinylation events in jujube, and there were more Kcr sites than Ksu sites, implying that Kcr was an abundant PTM in jujube and might play a vital role in substrate protein regulations.

### Motif characterization of lysine crotonylation peptides and local secondary structures

To investigate the sequence patterns of amino acids adjacent to the Kcr sites, the Motif-X program was used to observe the amino acid distribution from the -10 to + 10 positions around the Kcr sites (Fig. 2C). A total of thirteen clearly conserved motifs (with a motif score > 20) were identified (Additional file 6: Fig. S4A), namely, E**KcrK, K********KcrK, GKcrF, EKcrH, EKcrR, A*KcrE, E*KcrK, EKcrY, GKcrR, GKcrY, A**KcrK, KcrF*****K, and FKcrE. As shown in Fig. 2C, short aliphatic A residues were frequently observed in the -10 to + 10 position, positively charged K residue enrichment was observed in the -10 to -5 and + 5 to + 10 positions, and negatively charged residues D and E were markedly enriched in the -3 to + 3 positions. Notably, D and E were also found between peanut, tobacco, tea and papaya [11, 13, 14, 45], which were demonstrated to be extremely conservative and were rarely identified in other PTMs. The enrichment of E, K, R, F, Y and H was observed at the + 1 position, while A, D, E, F, G and Y enrichment was observed at the -1 position in jujube. These results showed that there may be a tendency for aromatic amino acids and aliphatic amino acids around crotonylation sites.

Furthermore, a conserved motif analysis of lys-succinylated peptides showed that only one motif of A*Ksu was found in jujube (Additional file 6: Fig. S4A). The intensity map showed that sites with K at the + 4, + 5, + 6, and + 8 positions and A at positions from -6 to -2 were more easily succinylated. However, K at the -1 and -2 positions showed the lowest frequencies. These results verified that there was a tendency for aliphatic amino acids near the succinylation sites in jujube (Fig. 2D). However, there was a tendency for heterocyclic, aromatic and aliphatic amino acids to exist around the succinylation sites in paulownia [9].

Structural analyses of all identified proteins indicated that 30% and 6% of the Kcr sites were located in the α-helix and β-strand, respectively, while 64% of the Kcr sites were located in disordered coils (Additional file 6: Fig. S4B). Compared with unmodified lysine residues, Kcr sites were found more frequently in the disordered coils (*P* = 6.8E-29) and less frequently in the α-helix (*P* = 5.7E-27) and β-strand (*P* = 3.1E-3) regions. Thus, it is clear that Kcr has a preference for secondary structures. The identified Kcr sites were further evaluated for solvent accessibility, and it was found that 37% of the Kcr sites were exposed to the protein surface, compared with 40% of those on unmodified lysine residues (*P* = 1.4E-43) (Additional file 6: Fig. S4B). In comparison with the unmodified counterparts, lysine residues at Kcr sites were less accessible on the surface. The lower surface accessibility of the Kcr sites implies that Kcr may occur in a selective process.

Succinylation sites tend to be distributed in unstructured regions (26% in α-helix, 6% in β-strand and 68% in unstructured regions) (Additional file 6: Fig. S4C), suggesting that PTMs in jujube tend to occur at coil regions, which is consistent with the results in other reports [9, 11]. Then, the distribution patterns of succinylated lysine residues and non- modified residues were compared, and the results showed that there was no preference for succinylation locations in jujube proteins (*P* = 0.17). A surface accessibility analysis revealed only a small decrease in the accessibility of succinylated lysine residues compared with non-modified residues (*P* = 0.86) (Additional file 6: Fig. S4C), indicating that Ksu did not alter the surface accessibility of jujube proteins.

### Functional enrichment of crotonylated and succinylated DEPs in jujube under phytoplasma stress

To explore the role of PTMs in jujube under phytoplasma stress, crotonylated or succinylated DEPs were analyzed (Table 1). It was found that 159 sites in 137 crotonylated proteins and 72 sites in 43 succinylated proteins were associated with phytoplasma presence. Remarkably, the downregulated modified proteins accounting for 75% of the total DEPs were quantified, suggesting that crotonylated modification and downregulated proteins may play significant roles in jujube during phytoplasma infection.

**Table 1:** The number of differentially abundant proteins and sites in diseased jujube

Comparison group (Diseased/Healthy)	Regulated type	Number
Whole protein	up-regulated	397
	down-regulated	259
Crotonylated protein (site)	up-regulated	42 (44)
	down-regulated	98 (115)
Succinylated protein (site)	up-regulated	3 (3)
	down-regulated	40 (69)

**Fig. 3:**
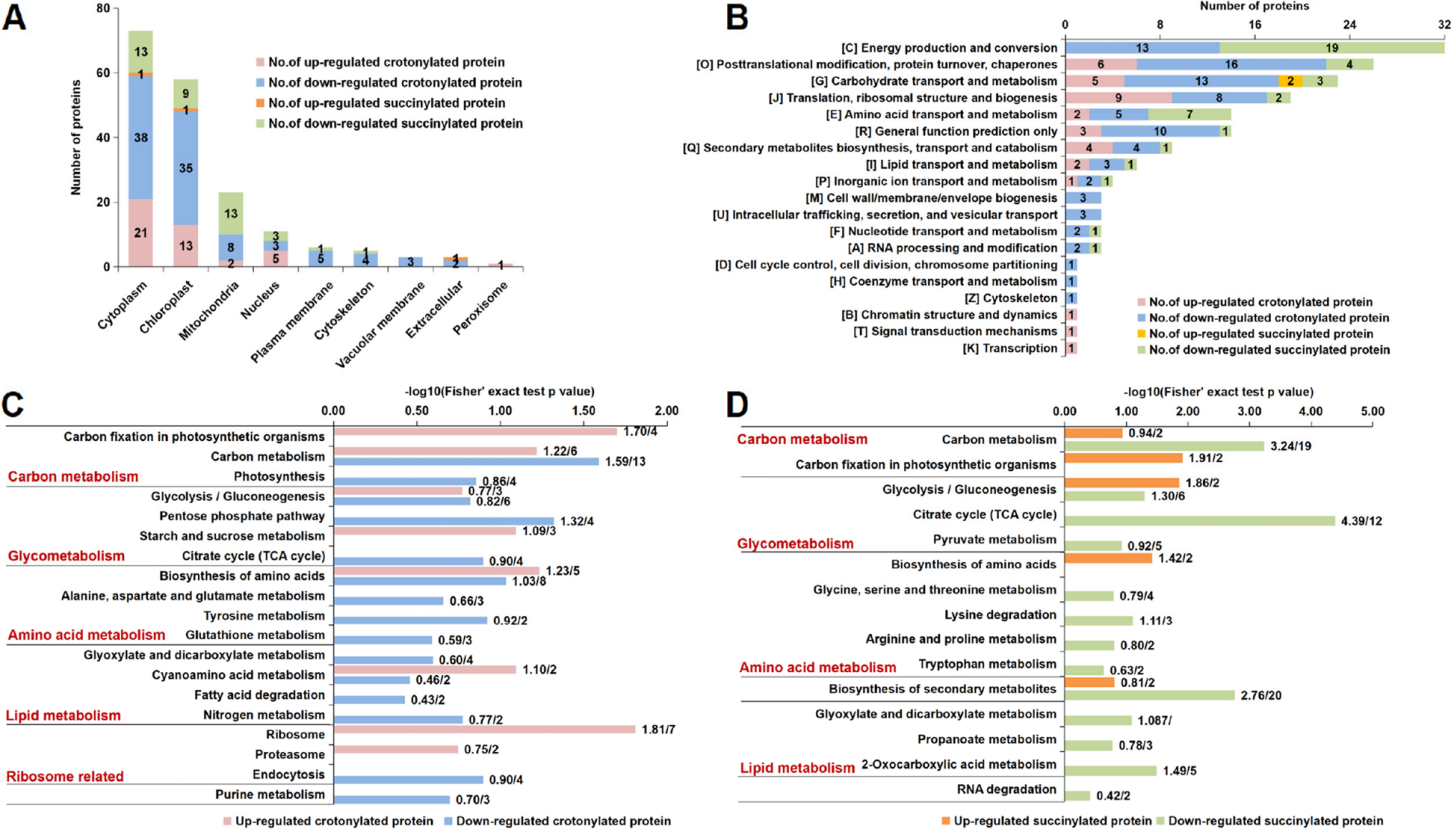
Functional enrichment analysis of crotonylated and succinylated DEPs in jujube under phytoplasma stress. **A** Predicted subcellular localization analysis of crotonylated and succinylated DEPs. **B** KOG functional classification chart of proteins corresponding to differentially expressed modification sites. **C** KEGG enrichment analysis of crotonylated DEPs. **D** KEGG enrichment analysis of succinylated DEPs. The negative logarithm of Fisher’s exact test *P* value is shown on the X axes. The number of proteins found in each category was provided after the score

To clarify the functions of these crotonylated and succinylated DEPs in jujube, subcellular localization, KOG functional classification, and KEGG pathway enrichment were performed. In the subcellular localization analysis, crotonylated DEPs were mainly distributed in the cytoplasm and chloroplasts, while succinylated DEPs were mainly located in the cytoplasm and mitochondria (Fig. 3A). These results suggested that crotonylation may affect metabolic processes and photosynthesis, while succinylation may be more related to oxidative phosphorylation and the TCA cycle.

A KOG analysis of these crotonylation and succinylated proteins was performed, and the results showed that these proteins were involved in energy production and conversion, carbohydrate transport and metabolism, carbohydrate metabolism, posttranslational modification, protein turnover, chaperones, ribosomal structure and biogenesis (Fig. 3B). The KEGG pathway enrichment also showed that the upregulated crotonylated proteins involved in ribosomes, carbon fixation and biosynthesis of amino acids, while the downregulated proteins were enriched in the carbon metabolism, pentose phosphate pathway, biosynthesis of amino acids and endocytosis (Fig. 3C), which was consistent with the above KOG results. The succinylated downregulated proteins mainly participated in the TCA cycle, carbon metabolism, the biosynthesis of secondary metabolites and 2-oxocarboxylic acid metabolism, three upregulated proteins were involved in glycolysis/gluconeogenesis, starch and sucrose metabolism and carbon fixation (Fig. 3D).

### Crosstalk analysis among the proteome, crotonylome, and succinylome in jujube

**Fig. 4:**
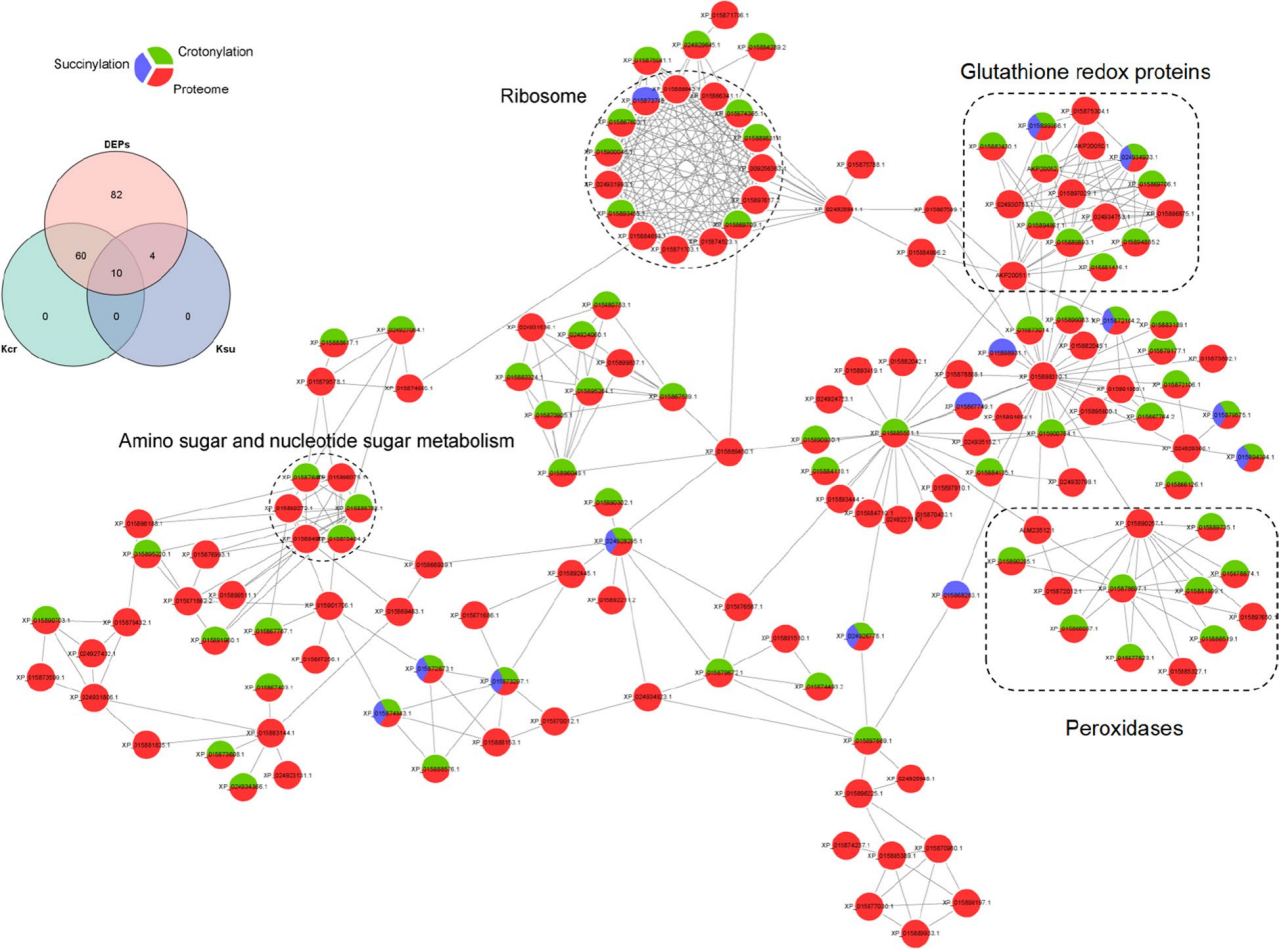
Crosstalk among JWB-related proteome, crotonylome and succinylome

There is growing evidence that crosstalk between the proteome and PTMs plays significant roles in regulating the function of non-histone proteins in plants and bacteria [46, 47]. Thus, a protein–protein interaction (PPI) analysis was conducted, and the network had 156 differentially expressed proteins, 70 crotonylated proteins and 14 succinylated proteins as nodes, which were connected by 406 direct physical interactions with a combined score higher than 0.70 (Fig. 4, Additional file 7: Table S3). In ribosome metabolism, 6 crotonylated proteins, 1 succinylated protein, and 8 nonacylated proteins were grouped, suggesting that they played key roles in protein synthesis. Three crotonylated and three nonacylated proteins participated in amino sugar and nucleotide sugar metabolism. Meanwhile, some redox enzymes were also grouped in this network and were mainly involved in glutathione metabolism and phenylpropanoid metabolism. Notably, 7 of the 8 peroxidase proteins involved in phenylpropane metabolism were identified as crotonylated proteins, such as peroxidase 51 (ZjPOD51). Meantime, crotonylation modification levels of several heat shock proteins were up-regulated, and proteins involved in glucose metabolism, amino acid and fatty acid metabolism were also enriched. And S-adenosylmethionine synthase 2 (ZjSAMS2), which involved in lipid metabolism, underwent crotonylation and succinylation at the same time. These specific proteins and their annotated functions were shown in Additional file 7: Table. S3.

**Fig. 5:**
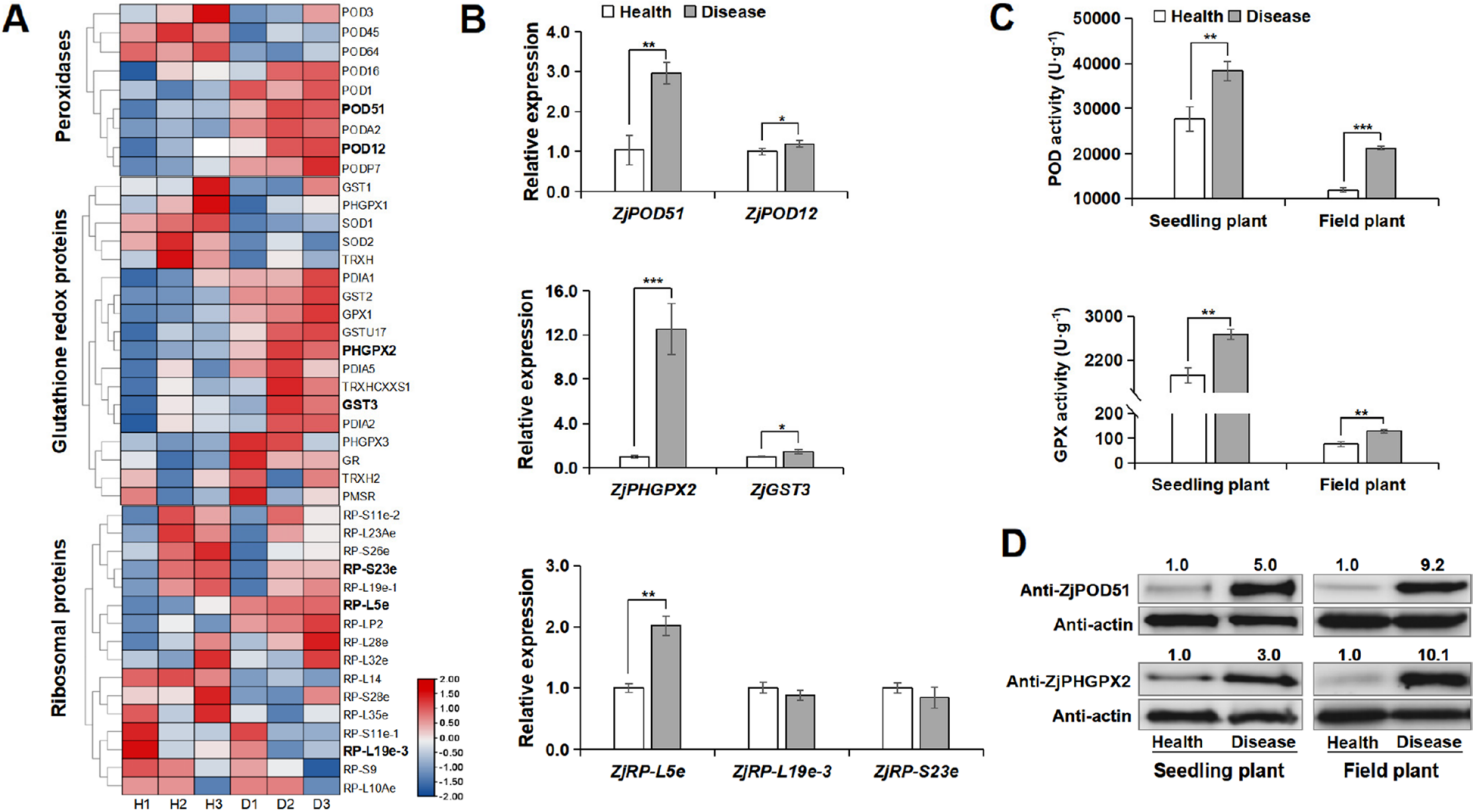
ZjPHGPX2 and ZjPOD51 were responsive to phytoplasma-stress. **A** Heat map analysis of DEPs related to ribosomal, peroxidase and glutathione redox in the healthy and diseased jujube trees. The accessions of proteins and their relative expressions in translation level were listed in Additional file 8: Table S4. **B** The transcriptional expressions of some DEPs by qRT-PCR. All data are presented as means ± SD from three independent experiments. Statistical significance was determined by independent t-test (*, *P* < 0.05; **, *P* < 0.01; ***, *P* < 0.001). **C** POD and GPX enzyme activities in healthy and diseased jujube trees (field) and seedlings (tissue culture). The raw data in Fig. 5B and 5C were shown in Additional file 9: Table S5. **D** WB analysis of ZjPHGPX2 (19 kDa) and ZjPOD51 (35 kDa) in healthy and diseased jujube trees (field) and seedlings (tissue culture) under phytoplasma stress. The original, uncropped gels/blots were shown in Additional file 10: Fig. S5

Next, the relative expressions of translation level showed that many ribosomal, peroxidases and glutathione redox proteins were truly up- or down-regulated in the diseased jujube trees (Fig. 5A, Additional file 8: Table S4), suggesting that these proteins are positively involved in the jujube-phytoplasma interactions. Correspondingly, the expressions of 7 related genes were also investigated (Fig. 5B). Among them, the expressions of two antioxidant genes, *ZjPHGPX2* and *ZjPOD51*, were significantly increased in diseased jujube. And the activities of the two enzymes were truly increased in both diseased jujube trees and diseased plantlets (Fig. 5C). Further, WB results also verified that ZjPHGPX2 and ZjPOD51 were highly expressed in diseased jujube (Fig. 5D). The results indicated that antioxidant proteins might play key roles in jujube-phytoplasma interaction, and some new candidate proteins undergo crotonylation at different lysine sites involved in the processes were identified (Additional file 4: Table S1, Fig. 4).

### Inhibitor NAM treatment reduces GPX enzyme activity

**Fig. 6:**
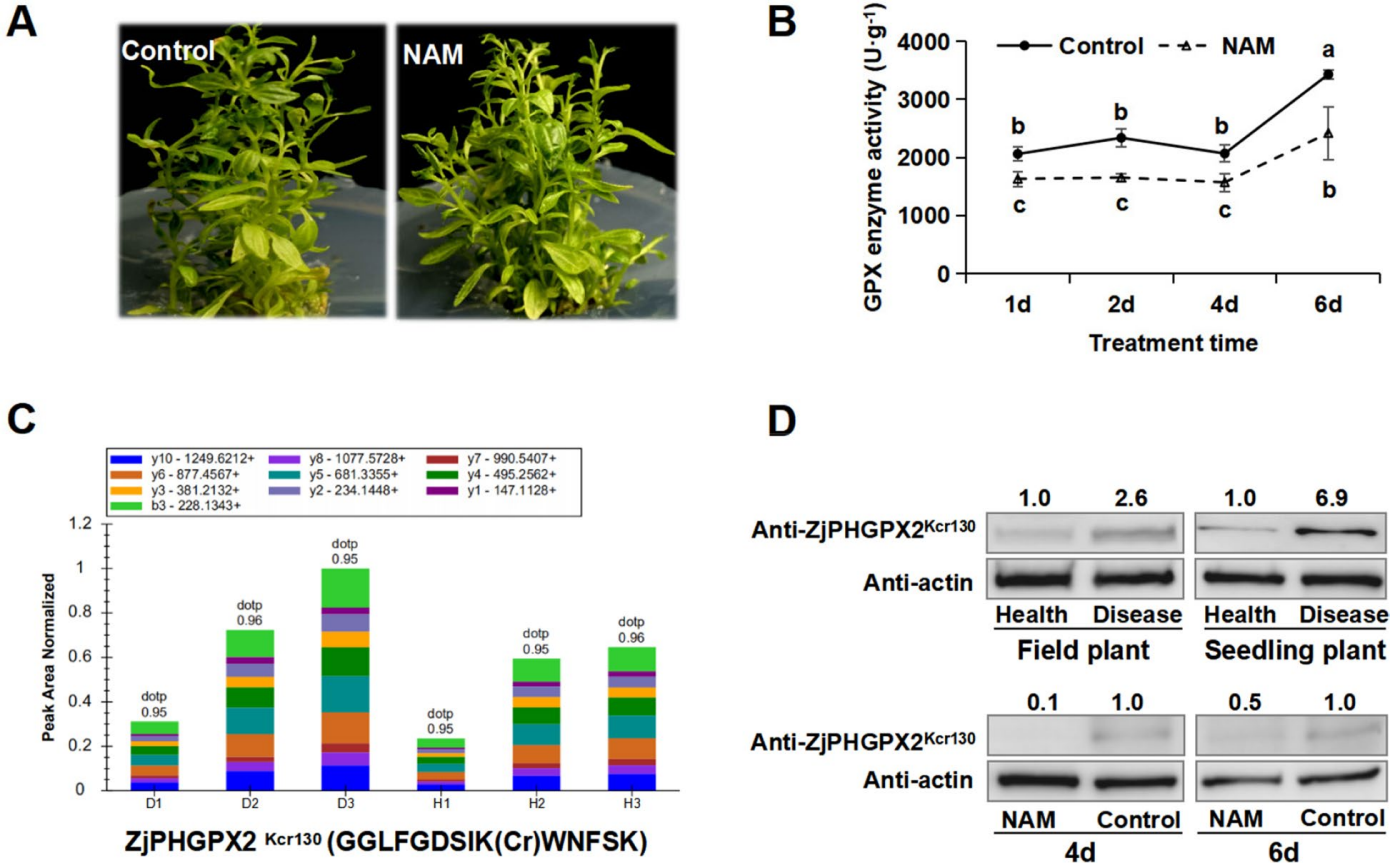
Inhibitors NAM significantly reduced the Kcr modification of ZjPHGPX2. **A** Phenotype of diseased seedlings after NAM treatment. **B** GPX enzyme activities of jujube seedlings after NAM treatment. The raw data were shown in Additional file 9: Table S5. **C** The diagram of ion peak area distribution of ZjPHGPX2^K130^ crotonylated peptide in healthy (H1, H2, H3) and diseased samples (D1, D2, D3). **D** WB analysis of ZjPHGPX2^Kcr130^ in healthy and diseased jujube trees (field) and seedlings (tissue culture), and diseased seedlings after NAM treatment. The original, uncropped gels/blots were shown in Additional file 11: Fig. S6

PHGPX was a unique antioxidant enzyme that markedly reduces lipid hydroperoxide generated in biomembranes. NAM was an inhibitor for sirtuin (SIRT) family deacetylases [48], and diseased jujube seedlings treated by NAM were investigated to determine whether Kcr in ZjPHGPX2 could be regulated by SIRT proteins. Compared with the control, the GPX enzyme activity significantly decreased in the diseased seedlings treated with NAM (Fig. 6B). PRM quantitative analysis verified that ZjPHGPX2 was lysine crotonylated at K130 (Fig. 6C). Therefore, a ZjPHGPX2^K130^ crotonylation specific antibody was constructed to detect its modification level. WB results showed that the crotonylation modifications of ZjPHGPX2^K130^ were increased in both diseased jujube plants. At the same time, the crotonylation modification levels of ZjPHGPX2^K130^ were indeed significantly reduced in jujube seedlings treated with NAM (Fig. 6D), indicating that inhibitor NAM treatment reduced GPX enzyme activity and crotonylation modification affected its activity, thereby altering its function in response to phytoplasma infection.

### Kcr modification at site K130 and K135 positively regulates ZjPHGPX2 activity

**Fig. 7:**
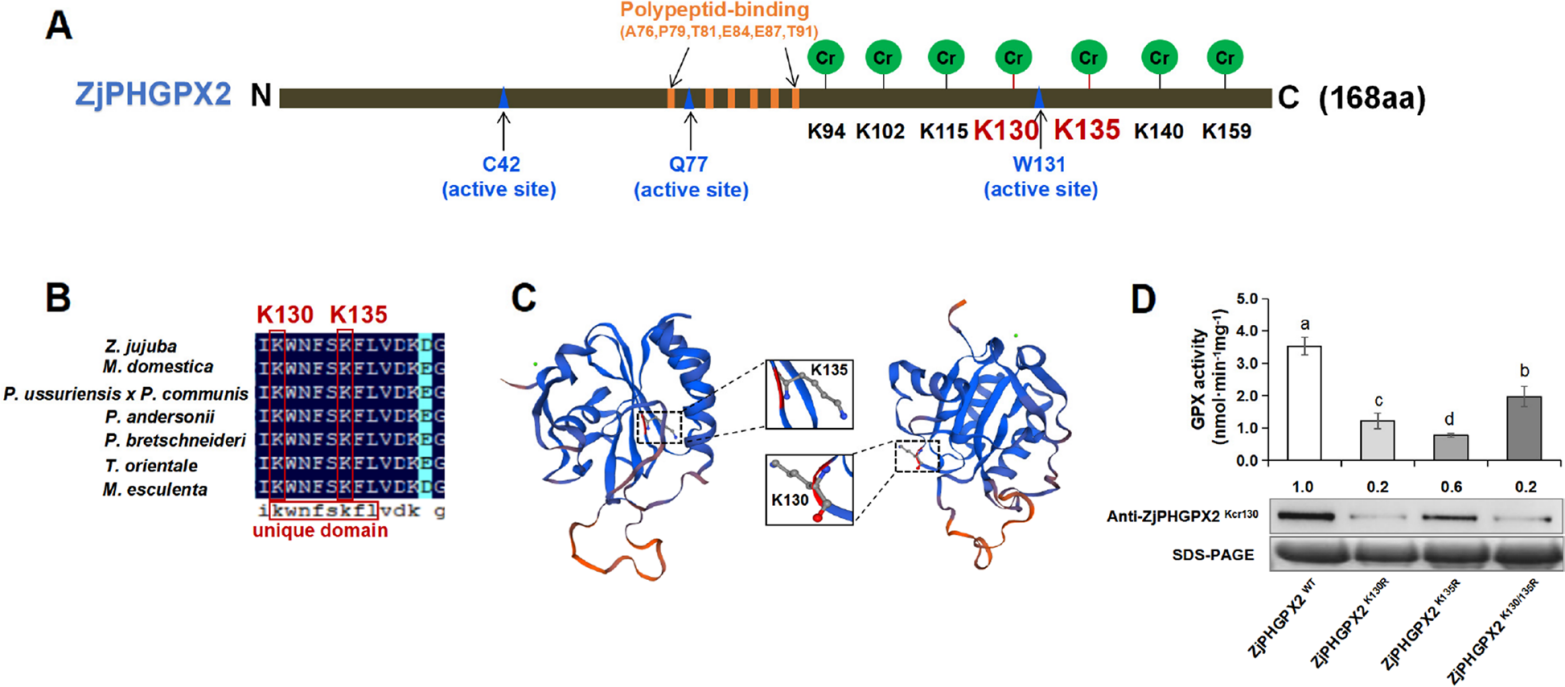
Kcr modification at site K130 and/or K135 positively regulates ZjPHGPX2 activity. **A** Location of Kcr sites in ZjPHGPX2. **B** Multiple sequence alignment of PHGPXs among jujube and other 6 species. **C** The tertiary structure prediction of ZjPHGPX2 constructed by SWISS-MODEL. **D** Effect of site-directed mutations at K130 and/or K135 of ZjPHGPX2 on GPX activity in *E. coli*. The different letters above the columns indicate significant differences according to Duncan’s multiple range test (*P* < 0.05). Values were presented as the mean of three replicates. The raw data were shown in Additional file 9: Table S5. WB analysis of ZjPHGPX2^Kcr130^ levels in different mutations. The original, uncropped gels/blots were shown in Additional file 14: Fig. S8

To further verify the Kcr sites of ZjPHGPX2, site-directed mutation was carried out. Based on domain analysis, ZjPHGPX2 contained a phospholipid hydroperoxide glutathione peroxidase conserved domain (PLN02399) consisting of a total of 156 amino acids from positions 9 to 165 (Additional file 12: Table S6), including 6 polypeptide-binding sites and 3 active sites (C42, Q77, W131) (Fig. 7A). Under phytoplasma stress, two key Kcr sites, K130 and K135, were identified in ZjPHGPX2, both sites were located near the activation site and within its protein specific domain KWNF (S/T) KFL. Sequence alignment showed that the K130 and K135 sites were evolutionarily conserved among various plant species and within the domain unique to PHGPX (Fig. 7B, C). Thus, site-directed mutations at K130 and/or K135 were performed, in which lysine was mutated to arginine (R) to induce decrotonylation. To investigate the effects of the mutations on ZjPHGPX2 function, the proteins of the wild-type and mutant forms were expressed and purified in *Escherichia* coli (Additional file 13, 14: Fig. S7, S8). The GPX activities in the mutations were significantly decreased compared to the wild-type. WB results further verified that the Kcr modification of ZjPHGPX2^K130^ in the mutants were significantly lower than that of wild-type (Fig. 7D). These results confirmed that the Kcr modification at K130 and/or K135 have positive impacts on the enzymatic activity of ZjPHGPX2.

## Discussion

Kcr is a newly discovered PTM that has been reported in several plants (Table 2). However, there is limited research on the modification of crotonylation in plants under stress conditions. In this study, Kcr in jujube, an important fruit tree in Asia, was first identified under phytoplasma stress. And the function of succinylation in paulownia-phytoplasma interaction was elucidated in a previous study [9]. Under phytoplasma-stress, the numbers of succinylated proteins in paulownia and jujube have an obvious dissimilarity (Table 2), meaning that the PTM could be species-specific. Compared to succinylation sites, more crotonylation sites were identified in jujube, indicating that crotonylation was an important PTM in jujube-phytoplasma interaction. In addition, phytoplasmas only colonize the phloem, this is an environment critical to their survival and multiplication. Thus, the phloem of jujube branches was used in this study, and the results could provide more direct clues to jujube-phytoplasma interaction.

**Table 2:** Comparison of proteome modifications among jujube and other plants

Acylation	No. of sites	No. of proteins	Plant species	Background	References
Lysine succinylation	605	262	*Brachypodium distachyon* L	No stress	[49]
	854	347	*Oryza sativa* L	Developing rice seeds	[19]
	3530	2132	*Camellia sinensis cv.‘Anji Baicha’*	Albinism	[21]
	259	202	*Carya cathayensis*	Grafting	[22]
	1970	1271	*Paulownia tomentosa*	Phytoplasma disease	[9]
	**570**	**282**	***Ziziphus jujuba***	**Phytoplasma disease**	**This study**
Lysine crotonylation	2044	637	*Nicotiana tabacum*	No stress	[11]
	1263	689	*Oryza sativa* L	No stress	[12]
	5995	2120	*Carica papaya* L	No stress	[13]
	6051	2508	*Arachis hypogaea* L	No stress	[14]
	5159	2272	*Broussonetia papyrifera*	No stress	[50]
	2017	1199	Chrysanthemum	Low temperature	[15]
	2288	971	*Camellia sinensis* L	NH^4+^ deficiency/resupply	[45]
	**3900**	**1656**	***Ziziphus jujuba***	**Phytoplasma disease**	**This study**

With respect to the subcellular localization of crotonylated proteins, the distributions reported for rice, papaya, tea, and tobacco were in the chloroplast, cytoplasm, nucleus, and mitochondria with the order of location frequencies. Under phytoplasma-stress, the top four locations in jujube were the cytoplasm, chloroplasts, mitochondria and nucleus. The cytoplasm, including ribosomes, storage, a variety of enzymes and intermediate metabolites, was the main place for plant metabolism. After the phytoplasma colonization, the ion environment in the cytoplasm of jujube cells was changed, and many metabolic processes were destroyed [42]. KEGG pathway enrichment analysis and the network of Kcr also showed that many ribosomal proteins were crotonylated. These findings suggest that Kcr played important role in jujube ribosome metabolism and that the regulation of ribosomal-related proteins were crucial for plants to cope with pathogens.

When plants were subjected to environmental or pathogen stress, ribosomes may affect protein synthesis and play an important biological role. The salicylic acid-related cotton ribosomal protein GaRPL18 contributes to the resistance of *Verticillium dahliae* [51]. When *Turnip mosaic virus* (TuMV) infected Arabidopsis and *Plum pox virus* (PPV) or *Tobacco mosaic virus* (TMV) infected tobacco, a large number of host ribosomal genes were upregulated [52, 53]. Further, ribosomal proteins can undergo different post-translational modifications, such as acetylation and phosphorylation [47, 54, 55], which may alter ribosome function. Significantly, *Magnaporthe oryzae* infection can promote oxidative modification of translation factors and ribosomal proteins in rice that are involved in protein translation [56]. These studies indicated that the regulation of ribosomal-related proteins was important for the interaction between plants and pathogens. After phytoplasma infection, a total of 31 ribosomal proteins were screened in jujube, of which 14 proteins with upregulated crotonylation sites were identified (Table 3). Whether the changes of ribosomal proteins expression and crotonylation level were involved in the interaction between jujube and phytoplasma needs further study.

**Table 3:** Ribosome-related proteins and crotonylation sites in jujube

Type	Protein accession	Protein description	Lysine crotonylation		Proteome	
			**D/H Ratio**	**Regulation**	**D/H Ratio**	**Regulation**
Ribosome subunits	XP_015894135.1	60S ribosomal protein L7-2 (cytoplasm)	0.799	Down	0.995	Normal
	XP_015894135.1	60S ribosomal protein L7-2 (cytoplasm)	33.913	Up		Normal
	XP_015902130.1	60S ribosomal protein L44 (nucleus)	9.203	Up	1.121	Normal
	XP_015892291.1	60S ribosomal protein L32-1(chloroplast)	0.758	Down	0.951	Normal
	XP_015866598.1	60S ribosomal protein L28-2-like (cytoplasm)	10.051	Up	1.125	Normal
	XP_024932225.1	60S ribosomal protein L26-1(cytoplasm)	0.759	Down	1.028	Normal
	XP_015877007.1	60S ribosomal protein L23A-like (nucleus)	1.229	Up	1.097	Normal
	XP_015891906.1	60S ribosomal protein L22-2-like (cytoplasm)	1.487	Up	1.112	Normal
	XP_015874352.1	60S ribosomal protein L11 (cytoplasm)	0.814	Down	0.949	Normal
	XP_015874365.1	40S ribosomal protein S23 (cytoplasm)	1.45	Up	0.787	Down
	XP_015874365.1	40S ribosomal protein S23 (cytoplasm)	39.346	Up		Down
	XP_015872987.1	60S ribosomal protein L21-1, partial (cytoplasm)	1.212	Up	0.961	Normal
Translation initiation factors	XP_015899905.1	translationally-controlled tumor protein homolog (cytoplasm)	0.776	Down	1.148	Normal
	XP_015899905.1	translationally-controlled tumor protein homolog (cytoplasm)	0.813	Down		Normal
	XP_015887772.1	eukaryotic translation initiation factor( mitochondria)	0.768	Down	1.029	Normal
Elongation factors	XP_015882124.1	elongation factor 2 isoform X1 (cytoplasm)	1.301	Up	1.093	Normal
	XP_015882124.1	elongation factor 2 isoform X1 (cytoplasm)	0.81	Down		Normal
	XP_015882124.1	elongation factor 2 isoform X1 (cytoplasm)	0.829	Down		Normal
	XP_015882124.1	elongation factor 2 isoform X1 (cytoplasm)	1.403	Up		Normal
	XP_015866981.1	elongation factor 1-delta 1-like (peroxisome)	1.209	Up	1.07	Normal
Aminoacyl-tRNA synthet	XP_015874032.1	lysine-tRNA ligase (cytoplasm)	0.293	Down	1.064	Normal
	XP_015872607.1	aconitate hydratase, cytoplasmic-like (cytoskeleton)	0.795	Down	1.143	Normal
Molecular chaperones	XP_015874072.1	heat shock cognate 70 kDa protein-like (cytoplasm)	0.701	Down	1.196	Normal
	XP_015876979.1	heat shock cognate 70 kDa protein 2-like (cytoplasm)	0.754	Down	1.033	Normal
	XP_015875846.1	stromal 70 kDa heat shock-related protein (chloroplast)	1.744	Up	0.972	Normal
	XP_015872929.1	temperature-induced lipocalin-1, partial (cytoplasm)	0.832	Down	1.381	Up
	XP_015874917.1	probable protein disulfide-isomerase A6 (vacuolar membrane)	0.828	Down	1.044	Normal
	XP_015874917.1	probable protein disulfide-isomerase A6 (vacuolar membrane)	0.121	Down		Normal
Proteasome subunits	XP_015896192.1	proteasome subunit beta type-6 (cytoplasm)	1.216	Up	0.936	Normal
	XP_015889725.1	proteasome subunit alpha type-7 (cytoplasm)	13.136	Up	1.028	Normal
	XP_015899007.1	26S proteasome regulatory subunit 8 homolog A (cytoplasm)	0.831	Down	1.013	Normal

Under pathogen attack, ROS in plants increase dramatically and balance of ROS production and clearance is destroyed, which leads to oxidative damage of proteins, nucleic acids, lipids [24, 25]. Then, the antioxidant defense system is triggered to protect the plant against the ROS, including GPX and POD, etc. [26]. In previous study, it was found that the enzymatic antioxidants including SOD, POD, laccase in jujube was triggered at transcriptional level by phytoplasma colonization [57]. Similar result was revealed at protein and Kcr levels in this study, indicating that enzymatic antioxidants truly play crucial roles in jujube at multiple levels to cope with phytoplasma colonization.

In previous study, overexpression of *OsPHGPX* improved the ability of rice to resist oxidative damage caused by paraquat [58], suggesting that *PHGPX* was involved in plant defense against oxidative stress caused by disease-stress. Scavenging ROS or enhancing antioxidant capacity effectively improved resistance to ferroptosis [59]. GPX4 SUMOylation at K125 site may affect the interaction between GPX4 and membrane phospholipids to regulate ferroptosis [60]. Lysine decrotonylation of DgGPX1 at K220 further increased GPX enzyme activity to reduce ROS accumulation under cold-stress, and thereby enhanced the cold resistance of chrysanthemum [16]. In this study, the levels of ZjPHGPX2 Kcr at K130/K135 in the unique domain (KWNF(S/T)KFL) were significantly increased under phytoplasma-stress. Site-directed mutation of key Kcr modification significantly reduced its activity. These results showed that the Kcr modification in GPX may function in opposite effects according to species or sites.

In summary, proteomics and PTM approaches were first used to survey changes in proteins and Kcr events in jujube-phytoplasma interaction. The results indicated that Kcr and Ksu of proteins occur extensively in jujube and that Kcr might be more widespread than Ksu in response to phytoplasmas. Further analysis found that some crotonylated proteins were associated with diverse biological processes, including photosynthesis, oxidative stress and protein biosynthesis, folding, and degradation. Moreover, some key DEPs and their Kcr modifications were screened and verified at multiple levels. Kcr modification at site K130 and/or K135 positively regulated ZjPHGPX2 activity. Overall, it provided a large-scale dataset of Kcr in phytoplasma-infected jujube and further elucidated the functional significance of crotonylated protein GPX in their interaction process.

## Conclusions

This study firstly provided large-scale datasets of Kcr and Ksu in phytoplasma-infected jujube. Kcr might be more widespread than Ksu in response to phytoplasmas. Further analysis found that some crotonylated proteins were associated with diverse biological processes, including photosynthesis, oxidative stress and protein biosynthesis, folding, and degradation. The crosstalk network among proteome, crotonylome and succinylome showed that DEPs related to ribosomal, peroxidases and glutathione redox were enriched. Among them, ZjPOD51 and ZjPHGPX2 significantly increased at the protein and Kcr level under phytoplasma-stress. After inhibitor NAM treatment, GPX enzyme activity in jujube seedlings was reduced. Further, site-directed mutagenesis of key Kcr modification sites K130 and/or K135 in ZjPHGPX2 positively regulates its activity.

## Methods

### Plant materials

In this study*,* the trees of *Ziziphus jujuba* Mill. ‘Pozao’ as field plant were cultivated in the Experimental Station of Chinese Jujube, Hebei Agricultural University. All experimental trees were cultivated under same conditions. The stem phloem samples were collected from three asymptomatic and three symptomatic trees infected with JWB on 14 June 2020. The annual secondary branches of jujube trees were selected, then the outermost layer in periderm tissue of these branches was peeled off, and the phloem was retained and collected (Additional file 15: Fig. S9). More than 10 g each sample was grinded into powder in liquid nitrogen, and stored at -80 °C for phytoplasma identification, modification omics analysis, qRT-PCR detection of DEPs, POD and GPX enzyme activity measurement, and WB detection. All treatments were conducted in triplicate.

The asymptomatic and symptomatic jujube seedlings of ‘Lizao’ were provided by Research Center of Chinese Jujube, Hebei Agricultural University. After subculture for 15 days, the seedlings with plant height of 4.0–5.0 cm were used for phytoplasma identification, POD and GPX enzyme activity measurement, WB detection, and inhibitor treatment. All treatments were conducted in triplicate.

### JWB phytoplasma detection

The 16S rDNA sequence is the most commonly used region in phytoplasma identification. The universal phytoplasma-specific primer sets P1/P7 (F: 5’-AAGAGTTTGATCCTGGCTCAGGATT-3’, R: 5’-CGTCCTTCATCGGCTCTT-3’) of 16S rDNA sequence was used for phytoplasma identification by PCR [61, 62]. The thymidylate kinase gene (TMK, KC493615.1) is the marker gene for JWB phytoplasma detection, which primer pair (F: 5’-GCAACAAATCCAAGAAGAGGAAA-3’, R: 5’-TTGCGAGGATAAGCTTGATAGG-3’) was used in this study [42]. The expression of the *TMK* gene in jujube samples exhibiting disease characteristics was analyzed by qRT-PCR with *ZjACT* as the internal control [63] (Additional file 1: Fig. S1).

### Enzyme activity analysis

A total of 0.1 g fresh samples were grinded into powder in liquid nitrogen and dissolved into 1 mL extraction buffer (BC1190, Solarbio, China). The mixture was centrifuged at 4 °C at 12,000 r.p.m for 20 min. Then the supernatant was sucked out on ice and the GPX enzyme activity was detected by measuring the decrease of NADPH absorbance at 412 nm according to the instructions of GPX assay Kit (BC1190, Solarbio, China). Similarly, the POD enzyme activity was determined according to the instructions of the POD enzyme activity assay Kit (BC0090, Solarbio, China). POD catalyzes H_2_O_2_ oxidation of specific substrates and has characteristic light absorption at 470 nm. The experiments were repeated three biological replicates.

### Protein extraction

The 0.5 g asymptomatic and symptomatic samples were quickly frozen with liquid nitrogen respectively, ground into powder with a frozen high-throughput tissue grinder (Scientz-48L), transferred to tubes, and then lysis buffer (8 M urea and 1% Protease Inhibitor Cocktail) was added. Protein extracts were treated according to the method reported by Cao et al. [9].

### Western blotting

First, proteins extracted by each sample mentioned above were separated on 12% SDS-PAGE gels. Then using a Trans-Blot Turbo transfer system (Bio-Rad, California, CA, USA), the above proteins were transferred to a polyvinylidine fluoride fluoropolymer (PVDF) membrane (0.45 µm, Millipore, Darmstadt, Germany) and then blocked with TBST (10 mM Tris–HCl, 0.05% Tween 20 and 150 mM NaCl, pH 8.0) including 5% BSA at 4 °C overnight. Finally, the 1:1000 dilution antibodies (PTM BioLabs, Hangzhou, China) were used for detection.

### Trypsin digestion, TMT labeling and HPLC fractionation

First, the proteins in solution were reduced with 5 mM dithiothreitol at 56 °C for 30 min and then alkylated with 11 mM iodoacetamide (Sigma) for 15 min in darkness at room temperature. Then, the proteins were diluted to urea concentrations lower than 2 M through adding 100 mM TEAB. Finally, the first digestion of protein with trypsin at a mass ratio of trypsin-to-protein of 1:50 was conducted overnight, and the second digestion with trypsin at a ratio of 1:100 was for 4 h.

After digestion, peptides were desalted by Strata X C18 SPE column (Phenomenex) and vacuum-dried. After reconstituting in 0.5 M TEAB, the peptides were processed for the TMT kit/iTRAQ kit. And one unit of TMT/iTRAQ reagent were thawed and reconstituted in acetonitrile (ThermoFisher chemical). Then the peptide mixtures were incubated for 2 h at room temperature and pooled, desalted and dried by vacuum centrifugation.

With an Agilent 300 Extend C18 column (250 mm length, 4.6 mm ID, 5 μm particles), the tryptic peptides were fractionated by high pH reverse-phase HPLC. The peptides were first separated with a gradient of 8% to 32% acetonitrile (pH 9.0) into 60 fractions over 60 min, and then combined into 18 fractions and dried by vacuum centrifugation.

### Affinity enrichment of the lysine crotonylated and succinylated peptides

To enrich the lysine-crotonylated and lysine-succinylated modified peptides, tryptic peptides were dissolved in NETN buffer (50 mM Tris–HCl, 100 mM NaCl, 1 mM EDTA, 0.5% NP-40, pH 8.0) and incubated with pre-washed antibody beads with gentle shaking overnight at 4 °C. Then, the beads were washed four times using NETN buffer and twice with ddH_2_O. The bound peptides were eluted from the beads with 0.1% trifluoroacetic acid and then combined and vacuum-dried. Finally, the resulting peptides were desalted with C18 ZipTips (Millipore) for the following LC–MS/MS analysis.

### LC–MS/MS analysis and database search

The LC–MS/MS analysis and database search were implemented according to the previous methods with slightly modification [9], for example, the electrospray voltage applied was 2.0 kV, the m/z scan range was 350 to 1800 for full scan in jujube. In paulownia, the m/z scan range was 350 to 1550. Tandem mass spectra were searched against the jujube transcriptome database. Carbamidomethyl on Cys was appointed as the fixed modification, and crotonylation or succinylation on lysine and oxidation on Met were appointed as variable ones.

For mass spectrometry-based targeted proteome quantification (PRM), the data were processed by using Skyline (v.3.6). The peptide settings were as follows: the enzyme was set as trypsin [KR/P], and max missed cleavage was set as 2. The variable modification was set as oxidation on Met and crotonylation on lysine, the maximum variable modifications were set as 3, and the peptide length was set as 8–25. The transition settings were as follows: the ion types were set as b, y, p, the precursor and ion charges were set as 2, 3 and 1, 2, respectively. The product ions were set as from ion 3 to the last one, and the ion match tolerance was set as 0.02 Da.

### Bioinformatics analysis

For GO annotation, the UniProt-GOA database (https://www.ebi.ac.uk/GOA/) was applied. Subcellular localization prediction was performed by Wolfpsort software. For pathway analysis, the KEGG database was used. KEGG annotated results were mapped via Mapper. Amino acid sequence motifs were screened through motif-X (http://motif-x.med.harvard.edu/). All the protein sequences obtained from the *Ziziphus jujuba* (common jujube) genome dataset in NCBI (https://www.ncbi.nlm.nih.gov/datasets/genome/GCF_000826755.1/), which were used as a background database parameter, and others were set to default values. Conserved domain analysis was conducted in NCBI. Secondary structures of protein surrounding the modified lysine residue were determined by using NetSurfP.

All differentially expressed modified protein sequences were searched against the STRING 10.1 for protein–protein interactions. All interactions with a confidence score ≥ 0.7 were selected. The interaction network was visualized in the R package “networkD3”.

### Analysis of differentially expressed modified and unmodified proteins

To compare the change in differentially expressed proteins between samples, the TMT quantification method and statistical analysis descried in Cao et al*.* was applied [9].

### Nicotinamide (NAM) treated jujube seedlings

The jujube symptomatic seedlings of ‘Lizao’ were cultured as described by Wang et al*.* [64]. After 15 days of subculture, the seedlings of 4.0–5.0 cm were used for NAM treatment (IN0150, Solarbio, China), with 10 mM NAM for 1d, 2d, 4d and 6d. NAM-treated seedlings were collected and then frozen in liquid nitrogen for enzyme activity measurement and WB assay. The plantlets treated by free water were used as control. GPX enzyme activity was assessed with a GPX assay Kit (BC1190, Solarbio, China). Each experiment was performed in triplicate.

### Total DNA, RNA extraction and qRT-PCR analysis

Total genomic DNA was extracted from field and seedling samples using the cetyltrimethylammonium bromide (CTAB) method for PCR to phytoplasma detection [65]. The PCR system and program were processed according to the methods reported by Ye et al. [61]. Total RNA was extracted using an RNAprep Pure Plant Kit (TIANGEN, Beijing, China). DNA and RNA concentration and purity were checked by a NanoDrop 2000 spectrophotometer. First strand cDNA was synthesized by 500 ng of the total RNA with a FastQuant RT Super Mix Kit (TIANGEN). cDNA was diluted tenfold and then used as the template for next study.

The expression levels of the genes texted were detected by qRT-PCR. The primers were listed in Table S4 and the amplicon sizes were within 80–250 bp. PCR products were amplified in triplicate using Bio-Rad iQTM5 with TransStart Top Green qPCR SuperMix AQ131 (TransGen Biotech, China) in 20 μL reactions. Three biological replicates were performed for each sample. To normalize the gene relative expression, *ZjACT* was co-amplified as the control [63]. Relative expression levels were calculated by the 2^−△△CT^ method. Healthy jujube plants were used as controls.

### Site-directed mutagenesis and prokaryotic expression

Conserved domain analysis of ZjPHGPX2 was performed in NCBI (https://www.ncbi.nlm.nih.gov/Structure/cdd/wrpsb.cgi). The amino acid sequence of ZjPHGPX2 was compared with the homologous sequences of other plants by DNAMAN. ZjPHGPX2 was subjected to whole-gene synthesis, and sites K130 and/or K135 were mutated to arginine (R). Complete de-crotonylation (K130R and/or K135R) was simulated according to the mutated charge stability. Three mutants and one non-mutant were selected by DNA sequencing. The coding sequences of ZjPHGPX2 and its Kcr site mutants were recombined into a prokaryotic expression vector [66–68], pETMAL-C2, using *EcoR* I and *Pst* I as restriction enzyme cutting sites. Gene synthesis and expression vector construction were completed by Genewiz Biotech (Suzhou) Co., Ltd. The recombined plasmids were transformed into *E. coli* BL21 (DE3) for protein expression. Using MBP beads (New England BioLabs), the recombinant maltose-binding protein (MBP)-ZjPHGPX2 protein and three mutates were purified. The collected PHGPX-WT purified recombinant proteins and other mutants were analyzed by SDS-PAGE and WB. The quantitative analysis of WB bands was performed by Image J software, and the data were homogenized.

The activity of the purified proteins were assayed with a GPX assay Kit (ADS-F-G003-48 Aidisheng Biotechnology, China) by measuring the decrease of NADPH absorbance at 412 nm in a coupled system. Each experiment was performed in triplicate.

### Antibodies

The anti-crotonyllysine antibody (Catalog No. PTM502), anti-succinyllysine antibody (Catalog No. PTM419), anti-2-hydroxyisobutyryllysine antibody (Catalog No. PTM801), anti-acetyllysine antibody (Catalog No. PTM101) used were commercially available antibody by PTM BioLabs (Hangzhou, China). Secondary anti-Mouse IgG, peroxidase antibody was used at 1:10,000 dilution. The anti-ZjPOD51 antibody (Catalog No. CM0309), anti-ZjPHGPX2 antibody (Catalog No. CM0307) and a specific anti-ZjPHGPX2^K130^ antibody (Catalog No. CK091501) were customized by PTM BioLabs. Anti-actin (plant) Mouse mAb (Catalog No. PTM6702, PTM BioLabs) was used as plant internal reference in this study.

### Statistical analysis

The data were presented as the mean ± SD of three independent experiments. Statistical analyses were conducted using one-way ANOVA with SPSS. Statistically significant differences were indicated by lowercase letters (*P* < 0.05). In addition, statistical analyses between two groups were analyzed by independent t-test and the significant differences were indicated by * (*P* < 0.05), ** (*P* < 0.01) or *** (*P* < 0.001).

### Supplementary Information

Additional file 1. Fig. S1. Identification and detection of phytoplasma in healthy and diseased jujube samples. (A, C) The universal phytoplasma-specific primer sets P1/P7 were used for phytoplasma identification in field and seedling samples by PCR, respectively. Note: M indicates DL 2000 marker, the 1.8 kb of destination bands were detected in the diseased samples, but not in the healthy plant. H_2_O was used as a negative control. (B, D) The thymidylate kinase gene (TMK, KC493615.1) was used for JWB phytoplasma detection in field and seedling samples by qRT-PCR, respectively. Note: n.e. indicates no expression. The raw data were shown in additional file 9: Table S5.

Additional file 2. Fig. S2. Coomassie brilliant blue staining of proteins in phloem of jujube.

Additional file 3. Fig. S3. The basic information of LC-MS/MS data. (A) The peptides score of LC-MS/MS data of crotonylation peptides. (B) Length distribution of all identified crotonylation peptides. (C) Box plot of RSD (Relative Standard Deviation) distribution of repeated samples using quantified crotonylation proteins. (D) The peptides score of LC-MS/MS data of succinylation peptides. (E) Length distribution of all identified succinylation peptides. (F) Box plot of RSD distribution of repeated samples using quantified succinylation proteins.

Additional file 4. Table S1. The information of crotonylated sites and their matched proteins.

Additional file 5. Table S2. The information of succinylated sites and their matched proteins.

Additional file 6. Fig. S4. Bioinformatics analysis of lysine crotonylation and succinylation sites. (A) Plot shows the relative abundance of amino acids flanking crotonylated lysine. The relative abundance was counted and schematically represented by an intensity map. The intensity map shows the enrichment of amino acids in specific positions of crotonylated lysine (10 amino acids upstream and downstream of the crotonylation or succinylation site). (B) Probabilities of Kcr in three different protein secondary structures (alpha-helix, beta-strand, and coli; left) and the predicted surface accessibility of Kcr sites (right). Lys: lysine. (C) Probabilities of Ksu in three different protein secondary structures (alpha-helix, beta-strand, and coli; left) and the predicted surface accessibility of Ksu sites (right). Lys: lysine.

Additional file 7. Table S3. Protein-protein interaction network of the JWB-related, crotonylated and succinylated proteins.

Additional file 8. Table S4. The primers information of DEPs and their relative expressions in translation level.

Additional file 9. Table S5. The relevant raw individual data points in this study.

Additional file 10. Fig. S5. WB analysis of ZjPHGPX2 and ZjPOD51 in healthy and diseased jujube trees (field) and seedlings (tissue culture) under phytoplasma stress. SH indicates healthy seedling plant; SD indicates diseased seedling plant; FH indicates healthy phloem of field plant; FD indicates diseased phloem of field plant; M indicates marker, the blue box indicates the destination strip.

Additional file 11. Fig. S6. WB analysis of ZjPHGPX2^Kcr130^ in healthy and diseased jujube trees (field) and seedlings (tissue culture), and diseased seedling plants after NAM treatment. SH indicates healthy seedling plant; SD indicates diseased seedling plant; FH indicates healthy phloem of field plant; FD indicates diseased phloem of field plant; M indicates marker, the blue box indicates the destination strip.

Additional file 12. Table S6. The conserved domain of ZjPHGPX2.

Additional file 13. Fig. S7. Purification of recombinant protein of ZjPHGPX2 and its mutants. (A-D) Optimization of purification conditions of recombinant protein of ZjPHGPX2 and its mutants. M indicates marker, the white box indicates optimal conditions for protein purification.

Additional file 14. Fig. S8. WB analysis of ZjPHGPX2^Kcr130^ levels in different mutations. (A) Coomassie brilliant blue staining of proteins in different mutations. (B) WB analysis of ZjPHGPX2^Kcr130^ levels in different mutations. M indicates marker, the blue box indicates the destination strip.

Additional file 15. Fig. S9. Schematic diagram of jujube branch.

## Data Availability

The data that supports the findings of this study are available in the supplementary material of the article. All the protein sequences obtained from the *Ziziphus jujuba* (common jujube) genomic dataset in NCBI (https://www.ncbi.nlm.nih.gov/datasets/genome/GCF_00082675 5.1/).

## Abbreviations

DEPs: Differentially expressed proteins
GO: Gene ontology
GPX: Glutathione peroxidase
GST: Glutathione S-transferase
JWB: Jujube witches’ broom
Kcr: Lysine crotonylation
KEGG: Kyoto Encyclopedia of Genes and Genomes
Ksu: Lysine succinylation
NAM: Nicotinamide
PHGPX: Phospholipid hydroperoxide glutathione peroxidase
POD: Peroxidase
PPI: Protein-protein interaction
PTM: Post-translational modification
PVDF: Polyvinylidine fluoride fluoropolymer
R: Arginine
ROS: Reactive oxygen species
RP-L5e: 60S ribosomal protein L5
TCA cycle: Tricarboxylic acid cycle
