# Supplementary figures and images for: The crotonylated and succinylated proteins of jujube involved in phytoplasma-stress responses
Source: BMC Biol. 2024 May 15;22:113. doi: 10.1186/s12915-024-01917-x (PMC11094900; doi:10.1186/s12915-024-01917-x)

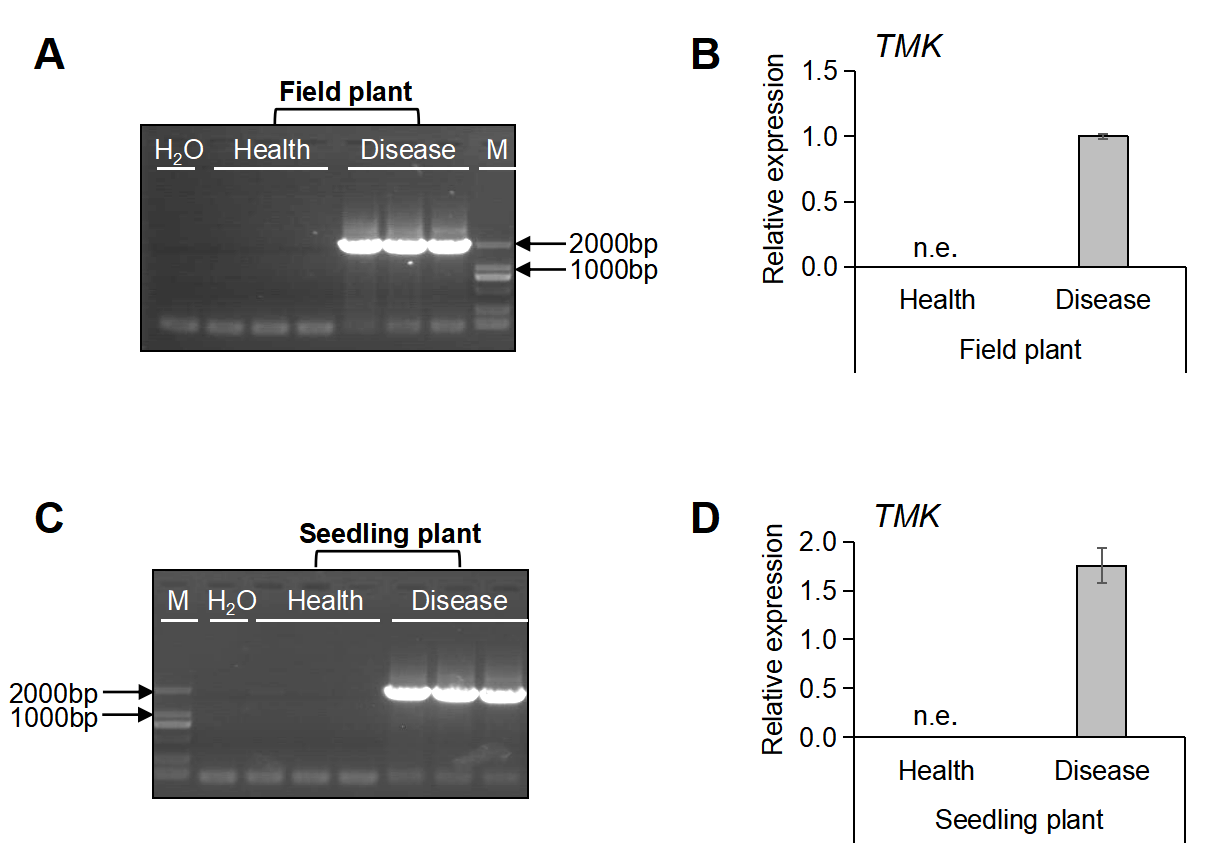

Supplement: Supplementary file 1 — Additional file 1. Fig. S1. Identification and detection of phytoplasma in healthy and diseased jujube samples. (A, C) The universal phytoplasma-specific primer sets P1/P7 were used for phytoplasma identification in field and seedling samples by PCR, respectively. Note: M indicates DL 2000 marker, the 1.8 kb of destination bands were detected in the diseased samples, but not in the healthy plant. H2O was used as a negative control. (B, D) The thymidylate kinase gene (TMK, KC493615.1) was used for JWB phytoplasma detection in field and seedling samples by qRT-PCR, respectively. Note: n.e. indicates no expression. The raw data were shown in additional file 9: Table S5. [file 12915_2024_1917_MOESM1_ESM.tif]

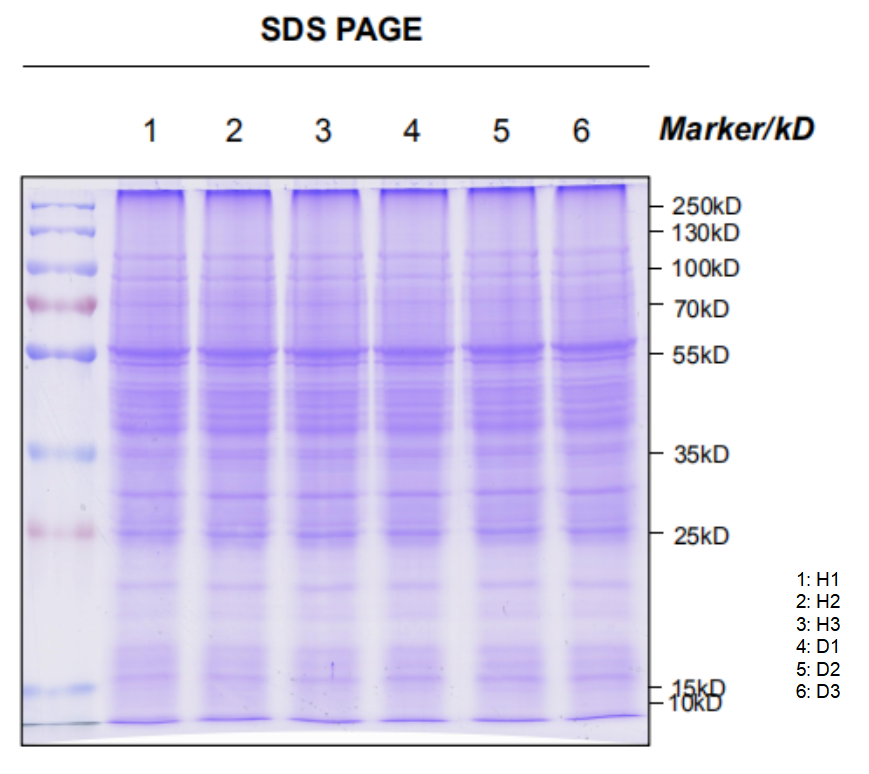

Supplement: Supplementary file 2 — Additional file 2. Fig. S2. Coomassie brilliant blue staining of proteins in phloem of jujube. [file 12915_2024_1917_MOESM2_ESM.tif]

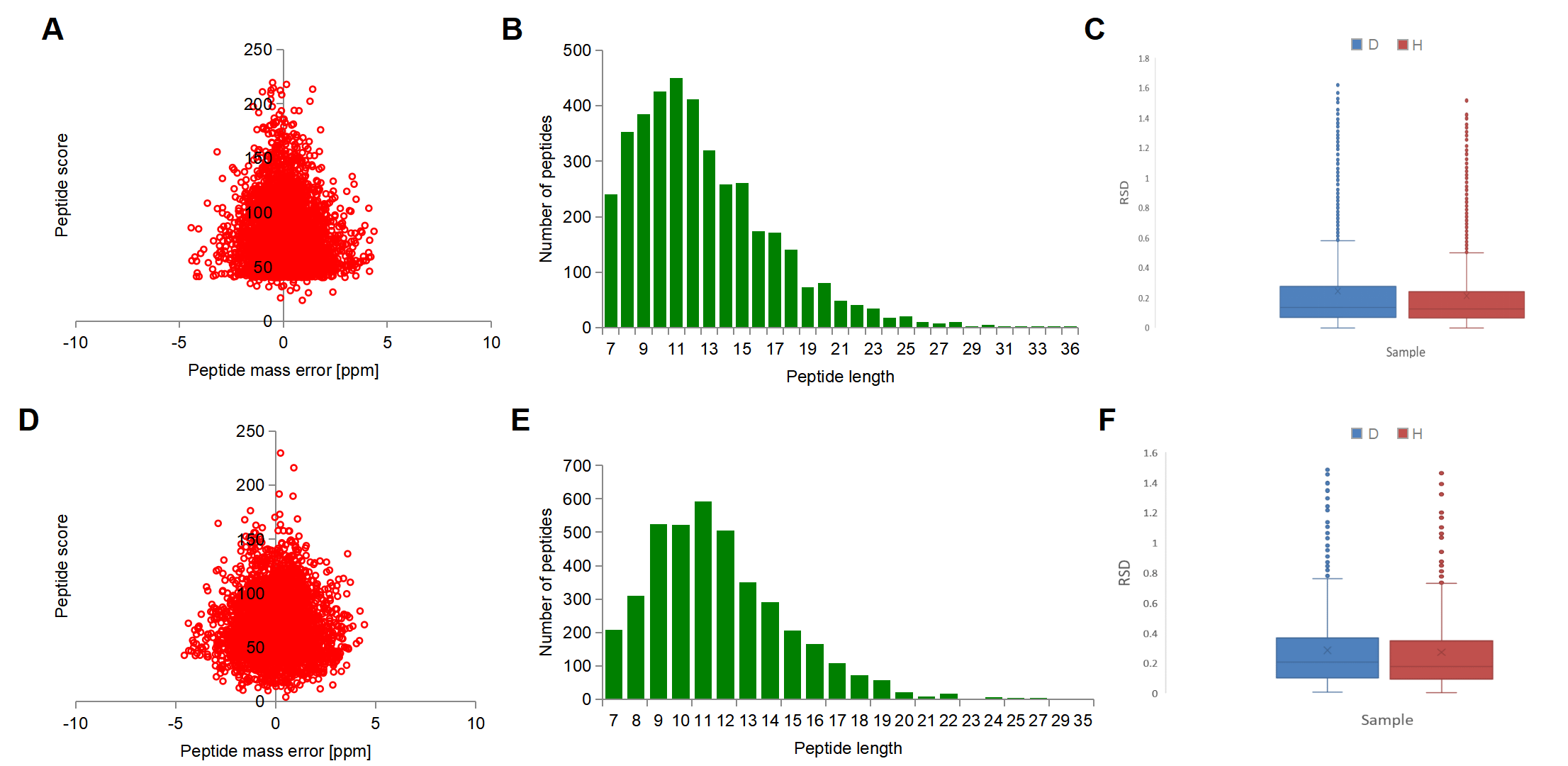

Supplement: Supplementary file 3 — Additional file 3. Fig. S3. The basic information of LC-MS/MS data. (A) The peptides score of LC-MS/MS data of crotonylation peptides. (B) Length distribution of all identified crotonylation peptides. (C) Box plot of RSD (Relative Standard Deviation) distribution of repeated samples using quantified crotonylation proteins. (D) The peptides score of LC-MS/MS data of succinylation peptides. (E) Length distribution of all identified succinylation peptides. (F) Box plot of RSD distribution of repeated samples using quantified succinylation proteins. [file 12915_2024_1917_MOESM3_ESM.tif]

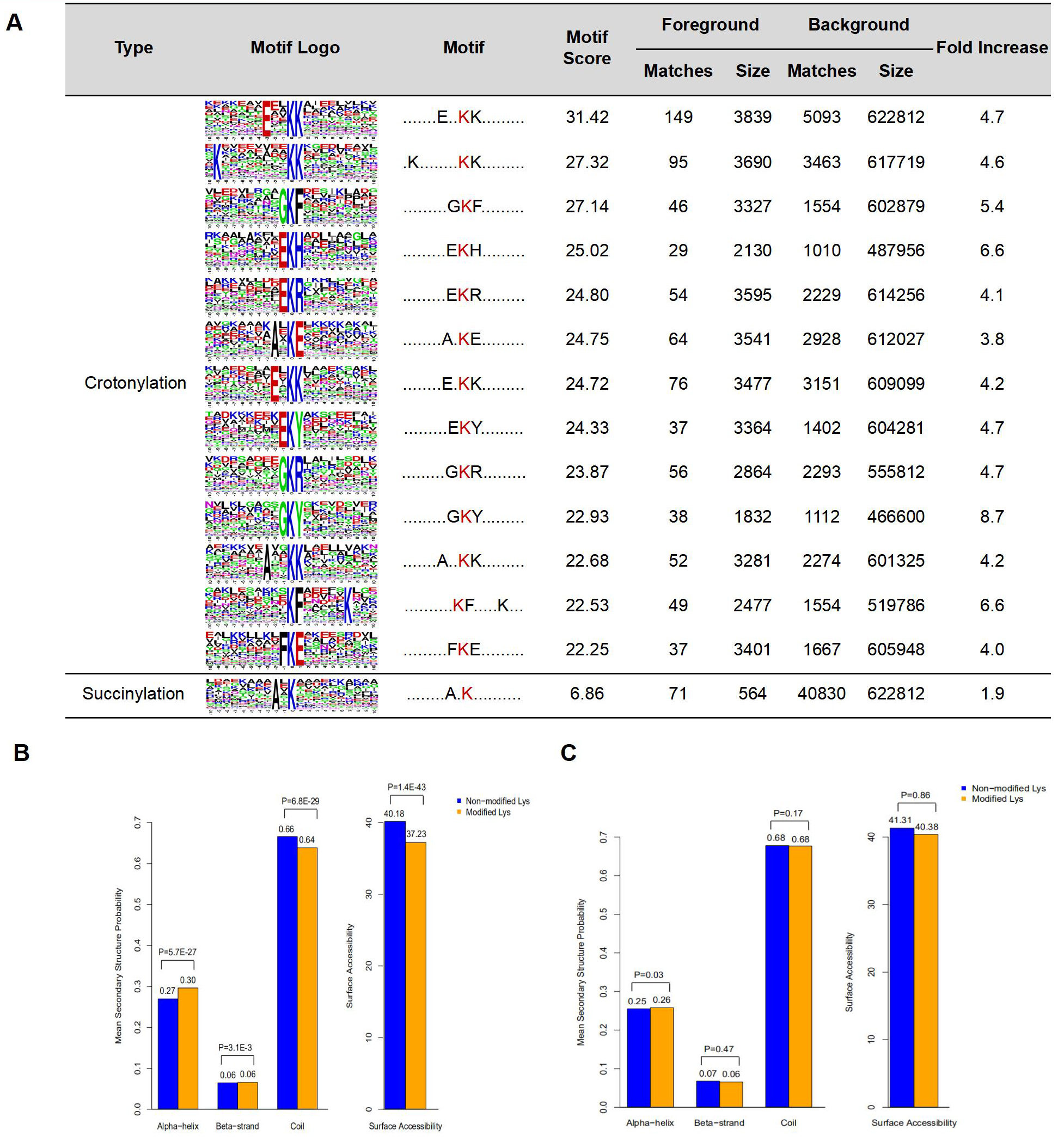

Supplement: Supplementary file 6 — Additional file 6. Fig. S4. Bioinformatics analysis of lysine crotonylation and succinylation sites. (A) Plot shows the relative abundance of amino acids flanking crotonylated lysine. The relative abundance was counted and schematically represented by an intensity map. The intensity map shows the enrichment of amino acids in specific positions of crotonylated lysine (10 amino acids upstream and downstream of the crotonylation or succinylation site). (B) Probabilities of Kcr in three different protein secondary structures (alpha-helix, beta-strand, and coli; left) and the predicted surface accessibility of Kcr sites (right). Lys: lysine. (C) Probabilities of Ksu in three different protein secondary structures (alpha-helix, beta-strand, and coli; left) and the predicted surface accessibility of Ksu sites (right). Lys: lysine. [file 12915_2024_1917_MOESM6_ESM.tif]

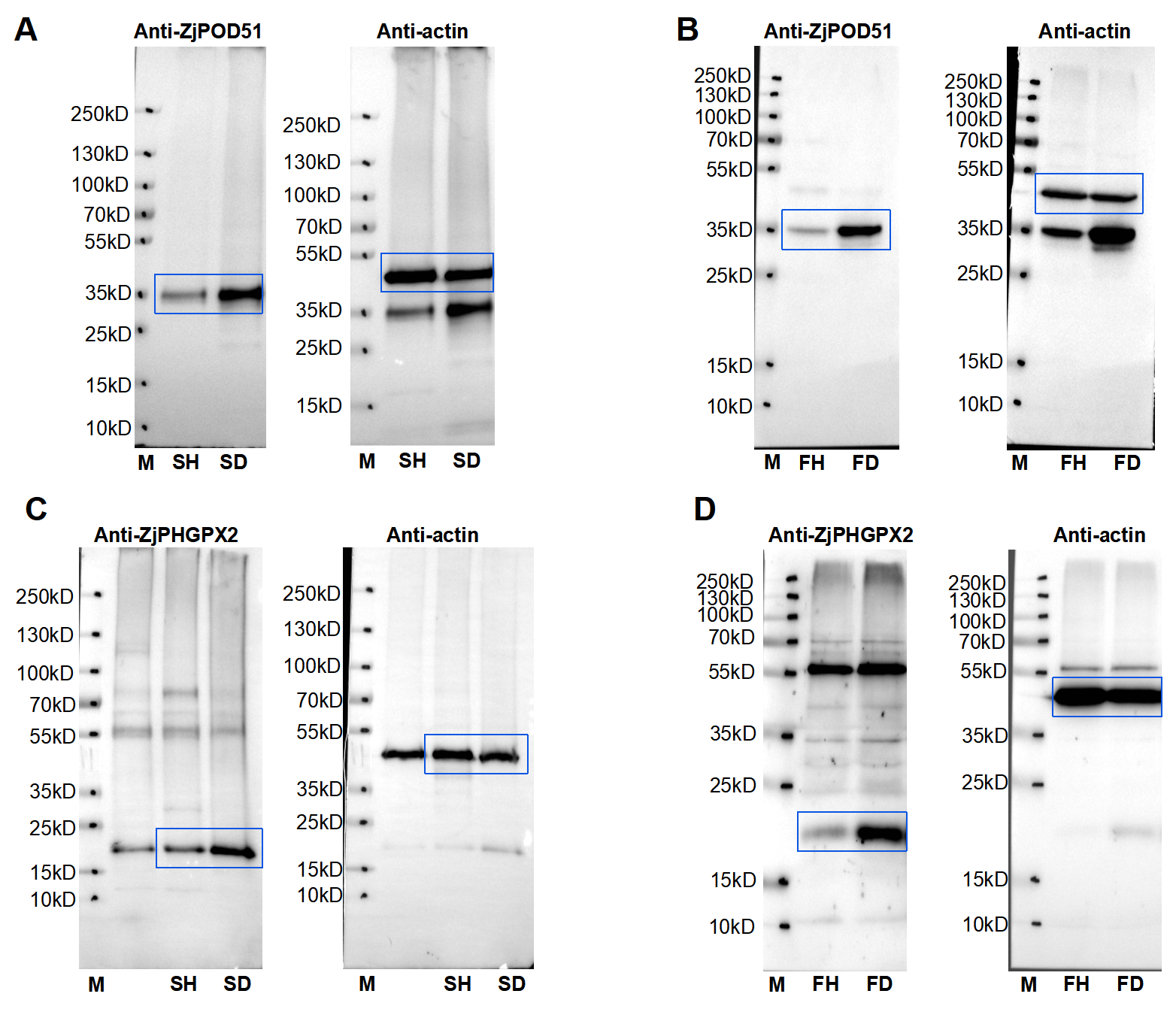

Supplement: Supplementary file 10 — Additional file 10. Fig. S5. WB analysis of ZjPHGPX2 and ZjPOD51 in healthy and diseased jujube trees (field) and seedlings (tissue culture) under phytoplasma stress. SH indicates healthy seedling plant; SD indicates diseased seedling plant; FH indicates healthy phloem of field plant; FD indicates diseased phloem of field plant; M indicates marker, the blue box indicates the destination strip. [file 12915_2024_1917_MOESM10_ESM.tif]

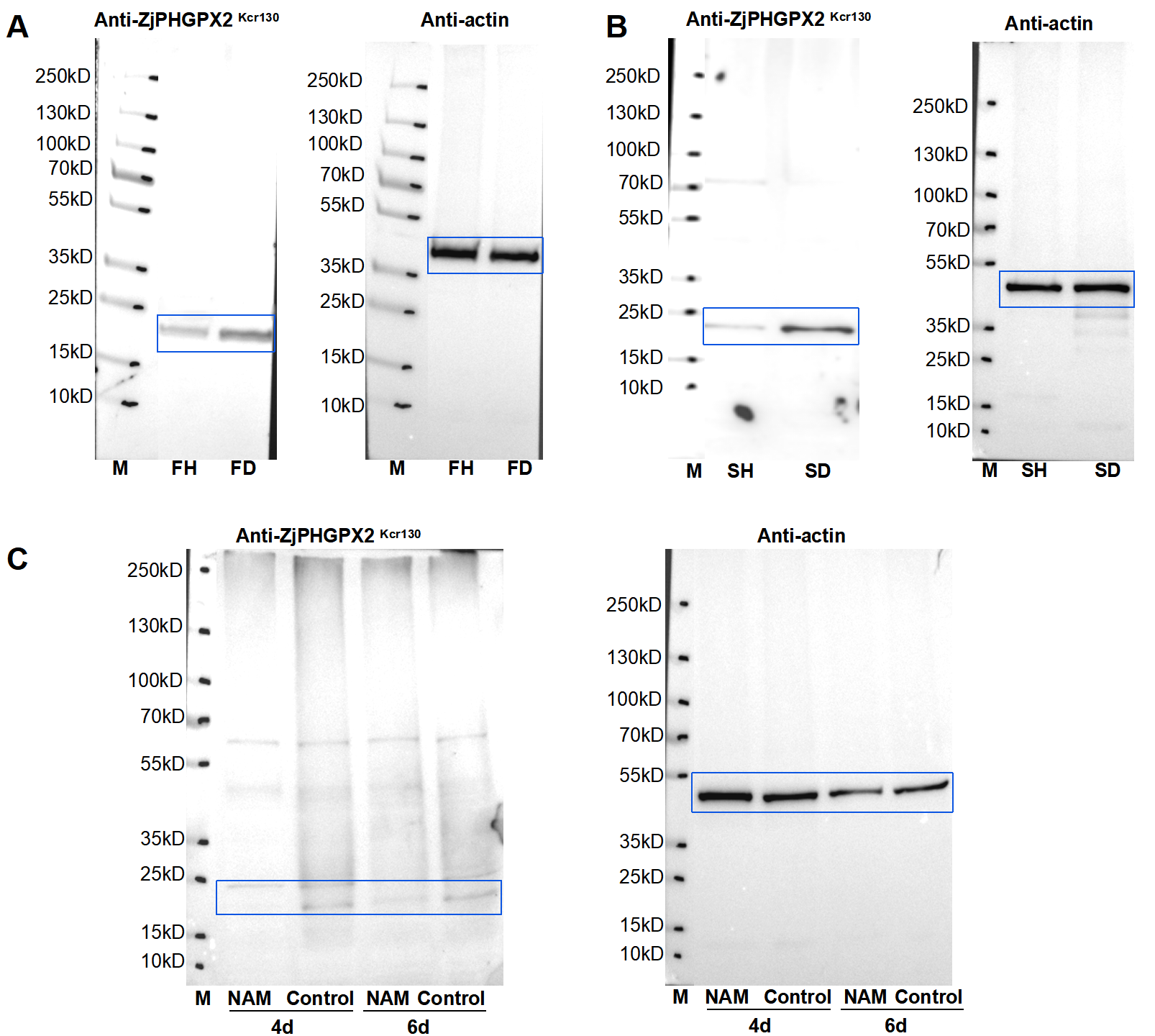

Supplement: Supplementary file 11 — Additional file 11. Fig. S6. WB analysis of ZjPHGPX2Kcr130 in healthy and diseased jujube trees (field) and seedlings (tissue culture), and diseased seedling plants after NAM treatment. SH indicates healthy seedling plant; SD indicates diseased seedling plant; FH indicates healthy phloem of field plant; FD indicates diseased phloem of field plant; M indicates marker, the blue box indicates the destination strip. [file 12915_2024_1917_MOESM11_ESM.tif]

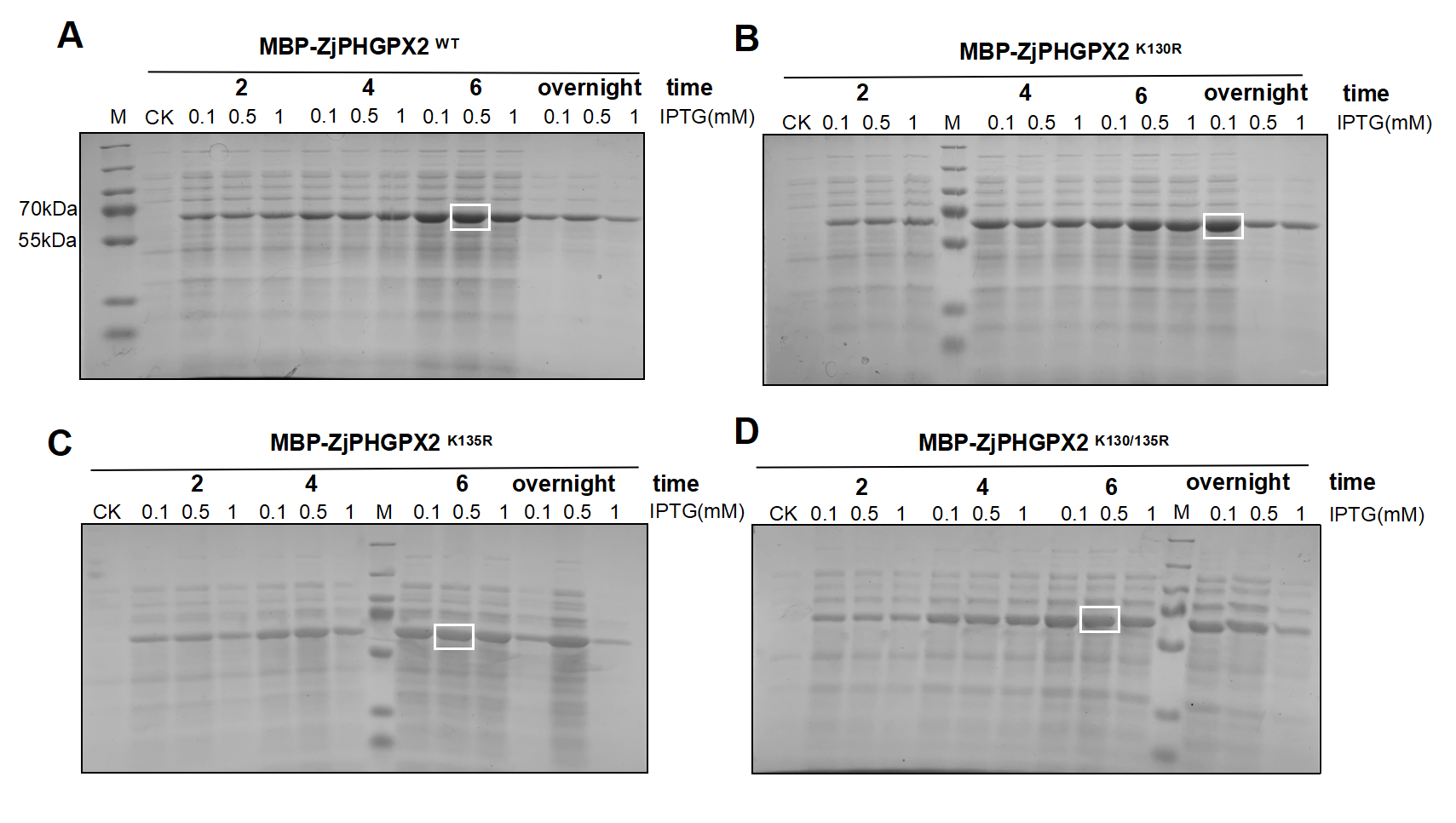

Supplement: Supplementary file 13 — Additional file 13. Fig. S7. Purification of recombinant protein of ZjPHGPX2 and its mutants. (A-D) Optimization of purification conditions of recombinant protein of ZjPHGPX2 and its mutants. M indicates marker, the white box indicates optimal conditions for protein purification. [file 12915_2024_1917_MOESM13_ESM.tif]

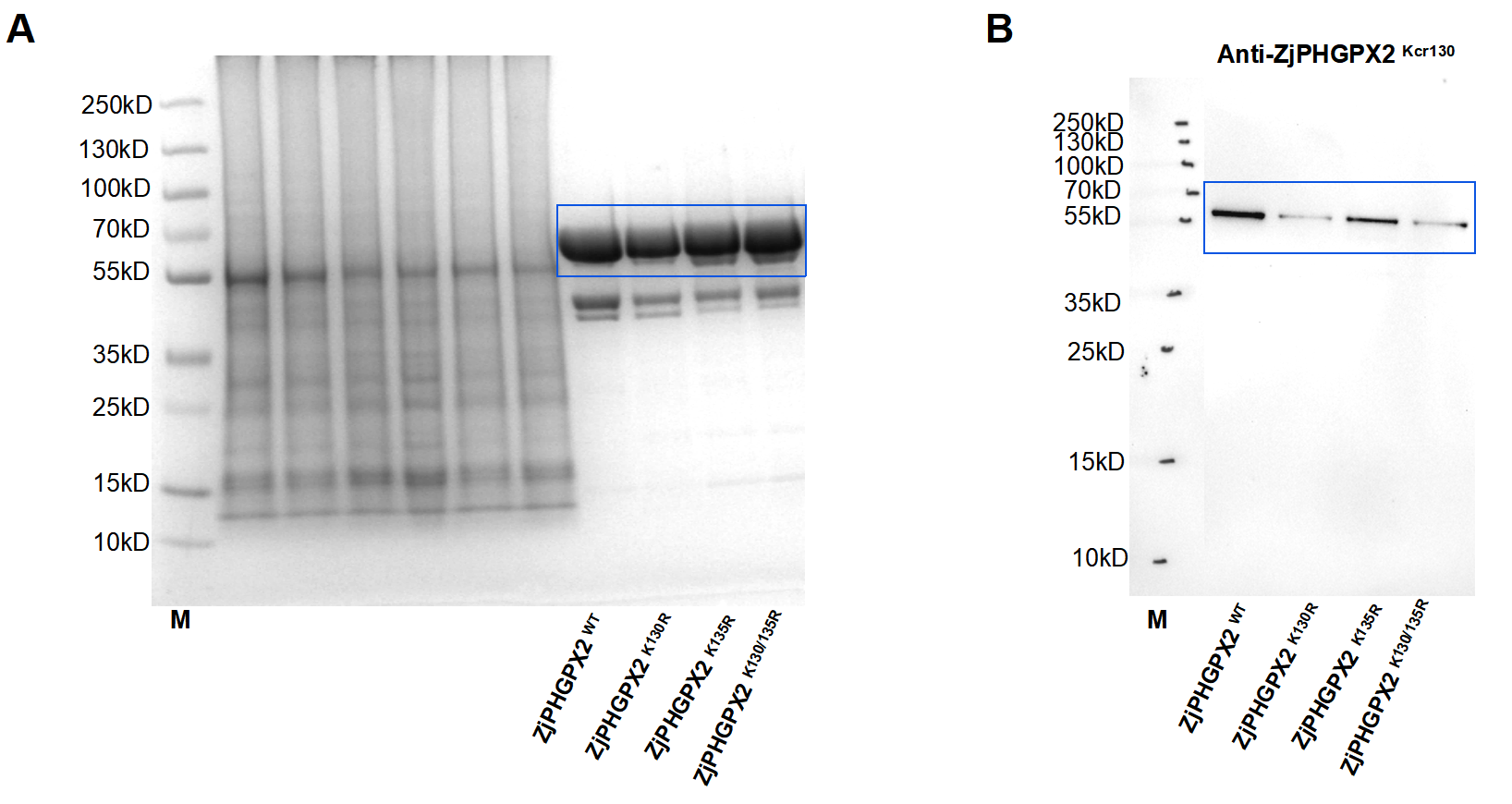

Supplement: Supplementary file 14 — Additional file 14. Fig. S8. WB analysis of ZjPHGPX2Kcr130 levels in different mutations. (A) Coomassie brilliant blue staining of proteins in different mutations. (B) WB analysis of ZjPHGPX2Kcr130 levels in different mutations. M indicates marker, the blue box indicates the destination strip. [file 12915_2024_1917_MOESM14_ESM.tif]

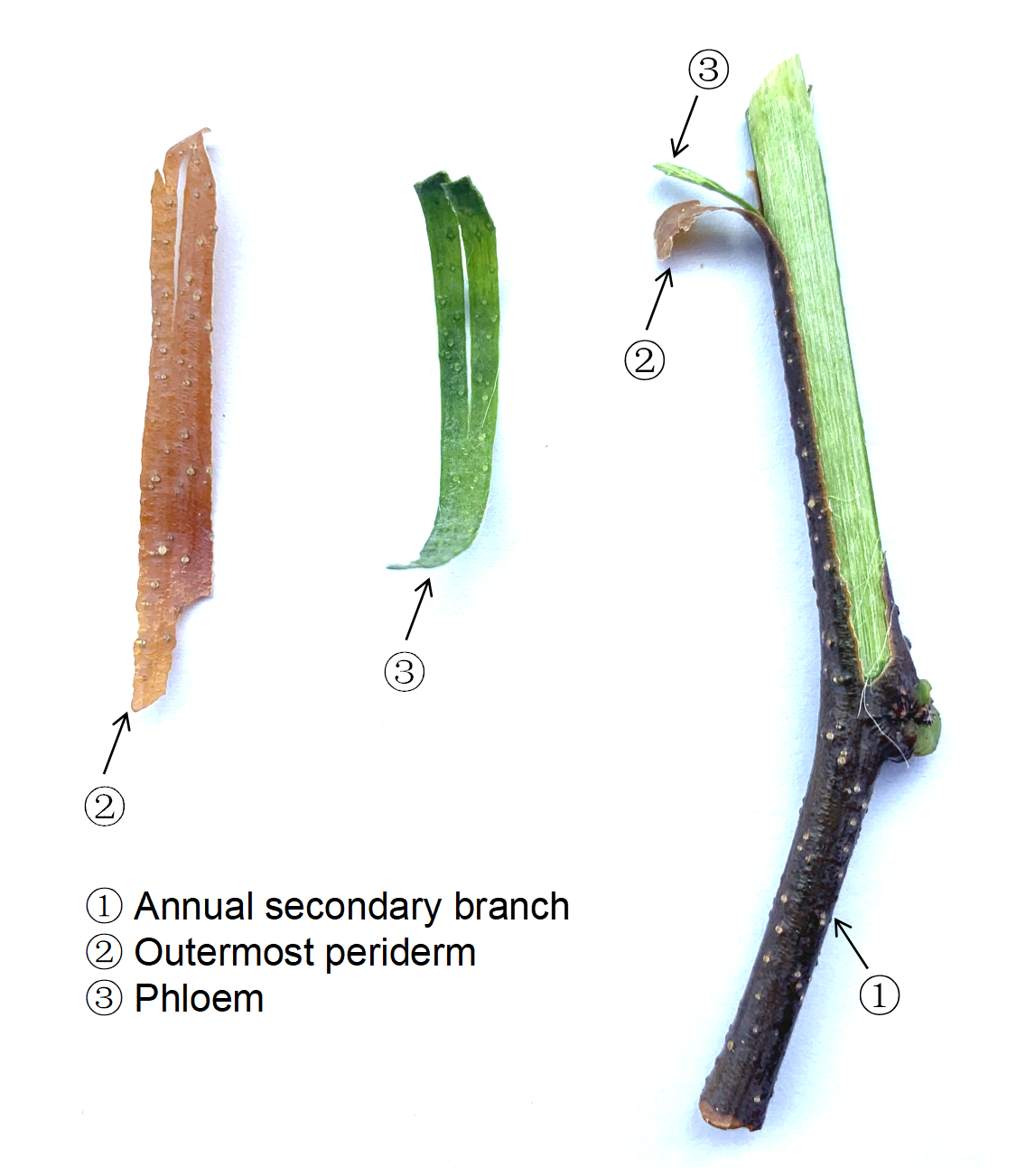

Supplement: Supplementary file 15 — Additional file 15. Fig. S9. Schematic diagram of jujube branch. [file 12915_2024_1917_MOESM15_ESM.tif]
